# Synthesis and biological evaluation of 3–(4-aminophenyl)-coumarin derivatives as potential anti-Alzheimer’s disease agents

**DOI:** 10.1080/14756366.2019.1615484

**Published:** 2019-05-22

**Authors:** Yu-Heng Hu, Jie Yang, Yun Zhang, Ke-Chun Liu, Teng Liu, Jie Sun, Xiao-Jing Wang

**Affiliations:** a School of Medicine and Life Sciences, University of Jinan-Shandong Academy of Medical Sciences, Jinan, PR China;; b Institute of Materia Medica, Shandong Academy of Medical Sciences, Jinan, PR China;; c Key Laboratory for Biotech-Drugs Ministry of Health, Jinan, PR China;; d Key Laboratory for Rare & Uncommon Diseases of Shandong Province, Jinan, PR China;; e Biology Institute, Qilu University of Technology (Shandong Academy of Sciences), Jinan, PR China

**Keywords:** Alzheimer’s disease, 3–(4-aminophenyl)-coumarin, acetylcholinesterase, butyrylcholinesterase, zebrafish

## Abstract

The work is focused on the design of drugs that prevent and treat Alzheimer’s disease (AD) and its complications. A series of 3–(4-aminophenyl)-coumarin derivatives designed, synthesised, fully characterised and evaluated *in vitro*/*vivo*. The biological assay experiments showed that some compounds displayed a clearly selective inhibition for acetylcholinesterase (AChE) and butyrylcholinesterase (BuChE). Among all compounds, compound **4m** exhibited the highest AChE inhibition with an IC_50_ value of 0.091 ± 0.011 µM and compound **4k** exhibited the highest BuChE inhibition with an IC_50_ value of 0.559 ± 0.017 µM. A zebrafish behaviour analyser (Zebrobox) was used to determine the behavioural effects of the active compound on the movement distance of the aluminium chloride-induced zebrafish. Compound **4m** offered a potential drug design concept for the development of therapeutic or preventive agents for AD and its complications.

## 1. Introduction

Alzheimer’s disease is a degenerative disease of the central nervous system that is mainly characterised by progressive memory impairment^1^^,^^2^. AD has brought heavy burdens to society and family because of the characteristics of progressive memory loss and loss of acquired knowledge^3^. The main characteristic pathological changes in the brain of patients with AD are the appearance of senile plaques (SPs)^4^ with extracellular amyloid β-protein (Aβ) deposition, and intracellular Tau hyperphosphorylation, formation of neurofibrillary tangles (NFTs)^5^, and loss of neurons^6^. Today there are 47 million people living with dementia worldwide. By 2050, this number will have increased above 135 million. Dementia affects 3.9 persons out of 1000 at the age ranging from 60 to 64^7^.

Although some work has been done on the pathogenesis of AD, there is no comprehensive and substantial cognition. The pathological theory widely accepted in the world is the “cholinergic hypothesis”. Acetylcholine is the neurotransmitter in the brain. The cholinergic hypothesis proposes that AD is caused by reduced synthesis of the neurotransmitter acetylcholine. ACh deficiency may cause learning loss and memory decline in patients. ACh levels and restoration of cholinergic nerve conduction can improve AD patients’ memory ability and cognitive level to reduce their symptoms. Therefore, increasing the ACh content in the brain of AD patients is a treatment for AD. AChE, a key enzyme in biological nerve conduction, can degrade ACh and terminate the excitatory effects of neurotransmitters on the postsynaptic membrane, which may be related to the formation of AD^8^. At present, there are no specific drugs for the treatment of AD. AChE inhibitors are currently the only ones that are universally recognised and have significant therapeutic effects in the world. They have a good effect on the treatment of mild-to-moderate AD. Among the mature AChE inhibitor drugs studied, there are mainly tacrine, donepezil, rivastigmine, galantamine^9^ and so on. Coumarin compounds are natural compounds with benzopyran ring nucleus. Previous studies showed that Schiff bases derivatives containing a triazole ring, uracil derivatives, aryl methanesulfonate derivatives are potent AChE and BChE inhibitors^10–12^. In addition to AChE, BuChE is important in the regulation of cholinergic system and it is reported to efficiently catalyse the hydrolysis of acetylcholine^13^. BuChE, an enzyme that breaks down artificial butyrylcholine, is known to hydrolyse ACh and other ester derivatives in the body^14^. BuChE, which is a tetrameric serine esterase consisting of monomers of ∼90-kDa molecular mass, showed over 65% structural similarity to AChE^15^. BuChE is expressed in a distinct population of neurons, some of which contain AChE. The importance of BuChE is further supported by the observation that AChE-knockout mice survive to adulthood indicating that BuChE is able to compensate for the lack of AChE, allowing the continued regulation of cholinergic neurotransmission^16^.

The drug’s efficacy depends on the interactions between the ligand and the biological target. The type of interactions affects the molecular mechanism of ligands inhibition of ChE activity. The research of new ChE inhibitory compounds is nowadays urgently needed^17^. Piazzi et al. ^18^ constructed a series of benzylpiperidine-coumarin derivatives by linking a benzylpiperidine moiety and a coumarin scaffold through a benzene ring. Here, we focus on the synthesis of 3–(4-aminophenyl)-coumarin derivatives and preliminary study of their pharmacological activities. This work has designed and synthesised a series of compounds and carried out a series of active screening around the research of anti-AD.

## 2. Results and discussion

### 2.1. Chemistry

**Scheme 1.**
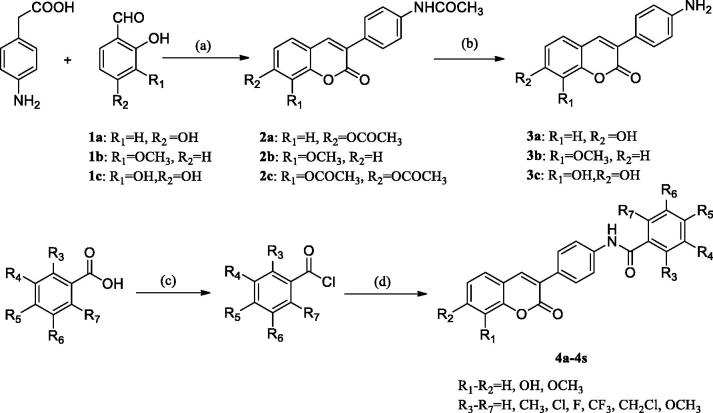
General synthetic route to 3–(4-aminophenyl)-coumarin **3a–3c** and compounds **4a–4s**. Reagents and conditions: a) acetic anhydride, Et_3_N, 115 °C; b) HCl ethanol; c) (COCl)_2,_ DCM, reflux; d) 3–(4-aminophenyl)-coumarin **3a–3c**, acetone, pyridine, and RT.

The synthetic route of the 3–(4-aminophenyl)-coumarin derivatives are summarised in (Scheme 1). Starting from substituted p-aminophenylacetic acids and substituted o-hydroxybenzaldehydes, **2a**–**2c** was obtained by Perkin reaction, followed by acidification with hydrochloric acid to give 3–(4-aminophenyl)-coumarin **3a**–**3c**. A series of substituted benzoyl chlorides were synthesised by reference methods and used in the next reaction^19^. The amide condensation of **3a**–**3c** with an acid chloride in a mixed solution of pyridine and acetone gives compounds **4a**–**4s** (Table 1). Details on the chemical and spectroscopic characterisations of compounds **4a**–**4s** were described in the Supporting Information.

**Table 1.** Compounds **4a–4s**, **5a–5b**, **6a–6b**, and **7a–7d**.

Product	R_1_	R_2_	R_3_	R_4_	R_5_	R_6_	R_7_	Yield (%)
**4a**	H	OH	H	F	H	H	H	66
**4b**	H	OH	H	H	F	H	H	70
**4c**	H	OH	H	H	CH_3_	H	H	58
**4d**	H	OH	H	H	CH_2_Cl	H	H	77
**4e**	H	OH	H	CH_3_	H	H	H	85
**4f**	H	OH	H	Cl	H	H	H	81
**4g**	H	OH	F	H	H	H	H	72
**4h**	H	OH	H	H	Cl	H	H	80
**4i**	H	OH	H	CH_3_	H	CH_3_	H	72
**4j**	OCH_3_	H	H	F	H	H	H	75
**4k**	OCH_3_	H	H	H	F	H	H	78
**4l**	OCH_3_	H	H	H	CF_3_	H	H	68
**4m**	OCH_3_	H	H	H	CH_2_Cl	H	H	70
**4n**	OCH_3_	H	H	Cl	H	H	H	81
**4o**	OCH_3_	H	H	CH_3_	H	H	H	87
**4p**	OCH_3_	H	F	H	H	H	H	82
**4q**	OCH_3_	H	OCH_3_	H	H	H	H	77
**4r**	OCH_3_	H	Cl	Cl	H	H	H	62
**4s**	OCH_3_	OH	H	H	CH_2_Cl	H	H	58
**5a**	H	OH	–	–	–	–	–	70
**5b**	OCH_3_	H	–	–	–	–	–	75
**6a**	H	OH	–	–	–	–	–	78
**6b**	OCH_3_	H	–	–	–	–	–	82
**7a**	H	OH	–	–	–	–	–	65
**7b**	OCH_3_	H	–	–	–	–	–	72
**7c**	OCH_3_	H	–	–	–	–	–	65
**7d**	OCH_3_	H	–	–	–	–	–	72

**Scheme 2.**
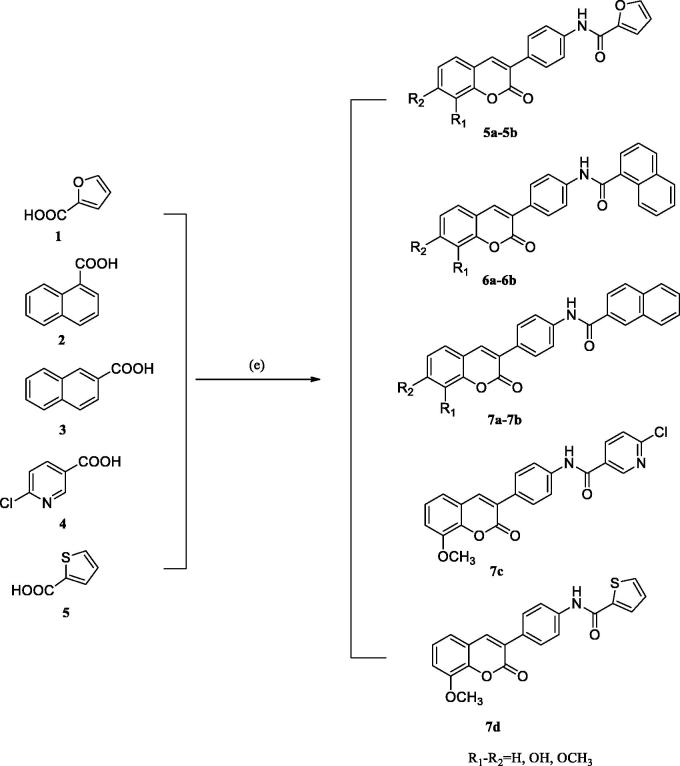
General synthetic route to compounds **5a–5b**, **6a–6b**, and **7a–7d**. Reagents and conditions: e) 3–(4-aminophenyl)-coumarin **3a–3b**, DCC, HOBt, toluene, and RT.

The synthetic route of 3–(4-aminophenyl)-coumarin derivatives bearing heterocycles is summarised in Scheme 2. Hydroxybenzotriazole (HOBt) and dicyclohexylcarbodiimide (DCC) was added into an anhydrous toluene solution containing compounds **1–6**. Then, compound **3a** or **3b** was added to the reaction solution, respectively. The reaction mixture was stirred at room temperature for 8–16 h to give compounds **5a–5b**, **6a–6b**, and **7a–7d** (Table 1). Details on the chemical and spectroscopic characterisations of compounds **5a–5b**, **6a–6b**, and **7a–7d** were described in the Supporting Information.

### 2.2. Biological evaluation

#### 2.2.1. *In vitro* cholinesterase inhibitory activity

AChE belongs to serine hydrolase family of enzymes catalysing the hydrolysis of neurotransmitter ACh into choline and acetic acid, consequently causing cessation of cholinergic neurotransmission^20^. AChE is widely distributed in conducting tissues like nerves and muscles, central and peripheral tissues, motor, and sensory fibres in addition to cholinergic and non-cholinergic fibres^21^. It is also present in plasma and blood cells^22^^,^^23^. As shown in Table 2, only eight compounds (**m**, **4f**, **4j**, **4 l**, **4o**, **5a**, **7 b**, and **7d**) presented moderate to excellent AChE inhibitory activity. Notably, **4 m** (IC_50_ = 0.091 ± 0.011 μM) had relatively strong activity, which was weaker than donepezil (IC_50_ = 0.012 ± 0.001 μM). The target compounds with an aromatic heterocyclic ring (such as compounds **5a**, **7b,** and **7d** have a furan ring, a naphthalene ring, and a thiophene ring, respectively) obtained by the amide condensation reaction and exhibited a good AChE inhibitory activity. Compounds **4d**, **4m**, and **4s** contain methyl chloride at the same position. However, compound **4m** with methoxy at R_1_ position has much better AChE inhibitory activity than compound **4s** with methoxy at R_1_ position and hydroxyl groups at R_2_ position. At the same time, the activity of compound **4m** is better than that of the compound **4d** contained hydroxyl groups at R_2_ position. Compounds **4a**, **4b**, **4c**, **4e**, **4g**, **4h**, and **4i** contained a hydroxyl group at R_2_ position and contained methyl, fluorine or chlorine, and these compounds have no inhibitory activity against AChE. The results indicated that compounds containing a methoxy group at R_1_ position and a methyl group, a fluorine atom or a chlorine atom (such as compounds **4g**, **4k**, **4m**, **4o**, **4p**, and **4q**), have a certain AChE inhibitory activity.

**Table 2.** Biological evaluation *in vitro*.

	IC_50_^c^ (μM)		
Compound	AChE^a^	BuChE^b^	SI^d^
**4a**	>50	3.885 ± 0.955	<0.097
**4b**	>50	2.445 ± 0.035	<0.050
**4c**	>50	15.65 ± 1.825	<0.874
**4d**	47.21 ± 3.566	8.905 ± 1.175	0.189
**4e**	>50	10.57 ± 0.132	<0.211
**4f**	1.944 ± 0.141	2.565 ± 0.275	1.319
**4g**	>50	24.45 ± 0.535	<0.489
**4h**	>50	3.881 ± 0.191	<0.078
**4i**	>50	2.265 ± 0.225	<0.045
**4j**	4.165 ± 0.161	0.905 ± 0.081	0.217
**4k**	27.97 ± 2.560	0.559 ± 0.017	0.020
**4l**	5.604 ± 0.132	4.325 ± 0.685	0.772
**4m**	0.091 ± 0.011	41.19 ± 1.044	452.637
**4n**	>50	0.951 ± 0.159	<0.020
**4o**	8.259 ± 0.581	>50	>6.054
**4p**	48.95 ± 2.325	15.96 ± 0.475	0.326
**4q**	48.21 ± 3.125	>50	>1.037
**4r**	19.68 ± 2.450	4.335 ± 0.125	0.220
**4s**	48.21 ± 2.685	>50	>1.037
**5a**	2.487 ± 0.151	9.555 ± 0.455	3.842
**5b**	47.12 ± 1.589	4.615 ± 0.055	0.098
**6a**	19.14 ± 0.981	>50	>2.612
**6b**	>50	>50	–
**7a**	>50	2.065 ± 0.045	<0.052
**7b**	0.128 ± 0.011	43.76 ± 1.965	341.87
**7c**	19.19 ± 1.640	3.381 ± 0.342	0.176
**7d**	6.631 ± 0.120	4.645 ± 0.245	0.700
Donepezil	0.012 ± 0.001	2.665 ± 0.015	222.08

^a^AChE from Electrophorus electricus.

^b^BuChE from equine serum.

^c^Each value represents the mean ± SD (*n*= 3).

^d^SI: selectivity index (IC_50_ BuChE/IC_50_ AChE).

In the human brain, BuChE is found in neurons and glial cells as well as in neuritic plaques and tangles in AD patients^24^. It has been recently reported that dual inhibition of AChE and BuChE might reduce the symptoms of AD owing to the key role of BuChE in hydrolysis of ACh^25^. As shown in Table 2, most of the compounds exhibited moderate to excellent inhibitory activity against BuChE. It is noteworthy that compounds **4b**, **4f**, **4i**, **4j**, **4k**, **4n**, and **7a** have relatively strong inhibitory activity, and their inhibitory activity is better than donepezil (IC_50_ = 2.665 ± 0.015 μM), especially compound **4k** (IC_50_ = 0.559 ± 0.017 μM). Through comparing the compounds **4a**, **4b**, **4f**, **4g**, **4 h**, **4j**, **4k**, **4p**, and **4r**, we find 3-arylcoumarins with an amino group can enhance its BuChE inhibitory activity by introducing halogen-containing benzene ring by the amide condensation reaction. We can find that the halogen-substituted compounds at the R_4_ and R_5_ positions are more active than the R_3_-substituted compounds by comparing their IC_50_ values. It can be seen that the halogen substitution position has a great influence on the inhibitory activity of BuChE.

According to *in vitro* AChE/BuChE inhibition test results, we decided to study the effects of compounds **4f**, **4j**, **4k**, **4l**, **4m**, **4r**, **5a**, **7b,** and **7d** on the aluminium trichloride-induced behavioural inhibition model of zebrafish larvae.

#### 2.2.2. Aluminium trichloride-induced behavioural inhibition model of zebrafish juveniles

AD causes the patient’s actions to become sluggish, and the zebrafish behavioural model can mimic AD from a motor perspective. AD model of zebrafish juveniles is produced by aluminium trichloride^26^^,^^27^. The zebrafish movement distance within 20 min was observed in a Zebrobox zebrafish behaviour analyser (Viewpoint, Lyon, France) by setting different concentrations of aluminium trichloride solution. The most suitable modelling concentrations were chosen, and then the effect of the compounds on the behaviour of the aluminium trichloride-induced zebrafish juveniles was explored. As shown in Table 3, we chose 5.0 mg/L aluminium trichloride as the best modelling concentration.

**Table 3.** Zebrafish movement distance (cm) at different concentrations of AlCl_3_ at 72 hpe (hours post-exposure).

Concentration (mg/L)	Control	0.1	0.5	1.0	5.0	10.0
Movement distance (cm)	290.2 ± 7.3	292.4 ± 6.7	236.8 ± 9.4*	224.3 ± 10.8**	181.3 ± 9.6**	204.6 ± 12.8**

Mean ± SE, *n* = 6, *p<.05, and **p<.01 compared to control.

The death and deformity of zebrafish were observed. As shown in Table 4, the blank group, 0.1, 0.5 mg/L group did not cause malformation and death of zebrafish for 3 days. Most of the fish in the 50.0 mg/L group were dead or malformed after 24 h administration; all fish died in the 50.0 mg/L group at 48 h, and malformed and dead zebrafish began in the 10.0 mg/L group; 5.0 mg/L groups of zebrafish have individual deaths and deformities after 72 h of administration.

**Table 4.** Effect of different concentrations of AlCl_3_ on mortality, deformity, and mortality of zebrafish juveniles after 72 h of administration.

Concentration (mg/L)	(Mortality rate (%)	Malformation rate (%)
Control	0.00 ± 0.00	0.00 ± 0.00
0.1	0.00 ± 0.00	0.00 ± 0.00
0.5	0.00 ± 0.00	0.00 ± 0.00
1.0	0.00 ± 0.00	1.11 ± 0.17
5.0	1.11 ± 0.17	2.22 ± 0.17
10.0	11.11 ± 0.17**	16.67 ± 0.29**
50.0	100.00 ± 0.00**	100.00 ± 0.00**

Each value represents the mean ± SE, (*n* = 90), *p<.05, **p<.01 compared to control.

#### 2.2.3. The effect of compounds on zebrafish model

**Figure 1.**
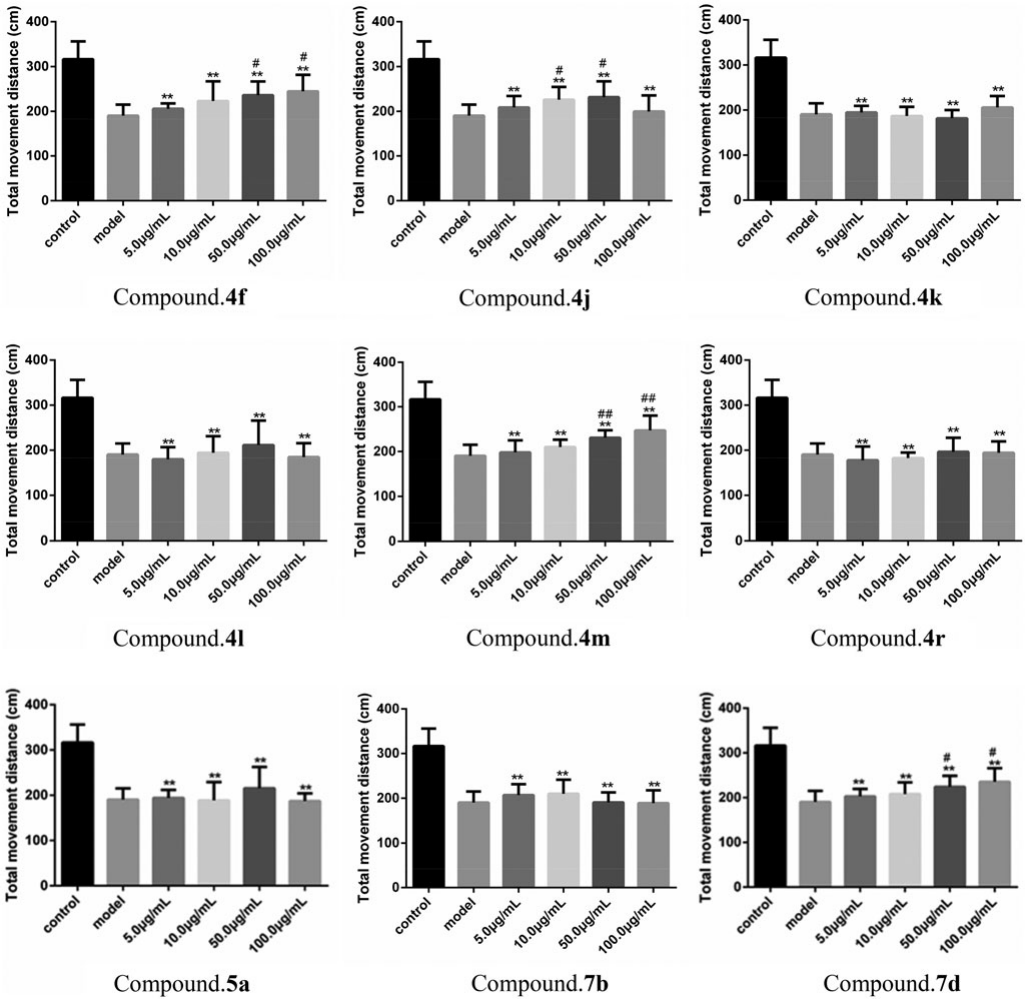
Effect of different compounds on the average total distance of zebrafish juveniles. *p < 0.05, **p<.01 compared to control; ^#^*p*<.05, ^##^*p*<.01 compared to the model group.

Zebrafish motion retardation characteristics were used to simulate motion disorders in AD. The main purpose of this experiment was to calculate the total distance of zebrafish movement induced by aluminium trichloride in the corresponding time after administration. As shown in Table 5, compounds **4f**, **4j**, **4m**, and **7d** have certain therapeutic effects on the behavioural inhibition of zebrafish induced by aluminium trichloride. In particular, compounds **4f**, **4j**, **4m**, and **7d** had significant effects compared with the model group. In the whole experiment, compound **4m** showed significant therapeutic effect at 50.0 and 100.0 μg/mL concentration, which could significantly increase the total distance of zebrafish juvenile movement induced by aluminium trichloride. With the increase of the concentration of compound **4j**, the total distance of zebrafish juvenile motion induced by aluminium trichloride increased first and then decreased. The total distance of zebrafish juvenile motion at the maximum concentration (100.0 μg/mL) was decreased compared to the total distance of zebrafish juvenile motion at 50.0 μg/mL. The results showed that high doses of compounds might slow down the movement distance of juvenile zebrafish. A more intuitive comparison can be seen in Figure 1.

**Table 5.** Effects of different compounds on the average total distance of zebrafish juveniles.

	Total movement distance (cm)					
	Control	Model	5 mg/L	10 mg/L	50 mg/L	100 mg/L
**4f**	316.4 ± 14.8	190.5 ± 9.3	205.7 ± 4.5**	223.2 ± 16.4**	236 ± 11.5^**#^	244.8 ± 13.6^**#^
**4j**	316.4 ± 14.8	190.5 ± 9.3	208.8 ± 9.6**	226 ± 10.7^**#^	231.8 ± 13.2^**#^	225.7 ± 13.4**
**4k**	316.4 ± 14.8	190.5 ± 9.3	194.9 ± 5.5**	186.7 ± 7.7**	181.8 ± 6.9**	205.7 ± 9.5**
**4l**	316.4 ± 14.8	190.5 ± 9.3	179.9 ± 10.1**	194.6 ± 13.6**	211.5 ± 20.2**	184.8 ± 11.5**
**4m**	316.4 ± 14.8	190.5 ± 9.3	198.3 ± 10.1**	210.2 ± 6.0**	230.8 ± 6.2^**##^	247.1 ± 12.3^**##^
**4r**	316.4 ± 14.8	190.5 ± 9.3	178 ± 11.2**	182.4 ± 4.6**	196.4 ± 11.7**	194.4 ± 9.4**
**5a**	316.4 ± 14.8	190.5 ± 9.3	194.2 ± 6.5**	188.8 ± 14.7**	215.5 ± 17.1**	187.2 ± 6.2**
**7b**	316.4 ± 14.8	190.5 ± 9.3	272.7 ± 8.9**	278.4 ± 11.4**	227.4 ± 8.1**	272.1 ± 10.4**
**7d**	316.4 ± 14.8	190.5 ± 9.3	202.9 ± 6.2**	208.4 ± 9.7**	224.2 ± 9.2^**#^	235.4 ± 11.4^**#^

Each value represents the mean ± SE, (*n* = 8), *p<.05, **p<.01 compared to control; ^#^*p*<.05, ^##^*p*<.01 compared to the model group.

## 3. Conclusions

Twenty-seven 3–(4-aminophenyl)-coumarin derivatives were synthesised by Perkin reaction, amide condensation reaction and carboxylic acid condensation reaction. We then studied the pharmacological activity of the synthesised compounds. The biological evaluation showed that most of the novel compounds were good dual cholinesterase inhibitors in the micromolar range. In particular, compound **4m** showed the highest effective AChE inhibitory activity (IC_50_ = 0.091 ± 0.011 μM), which was slightly weaker to the positive drug donepezil (IC_50_ = 0.012 ± 0.001 μM). The inhibitory activities of compounds **4b**, **4f**, **4i**, **4j**, **4k**, **4n,** and **7a** on BuChE were higher than the positive drug donepezil (IC_50_ = 2.665 ± 0.015 μM). Especially, compound **4k** showed the highest effective BuChE inhibitory activities (IC_50_ = 0.559 ± 0.017 μM), which 5-times more active than donepezil (IC_50_ = 2.665 ± 0.015 μM). Compounds **4 m**, **4o**, and **7b** were highly selective for AChE and compounds **4a**, **4 b**, **4h**, **4i**, and **4k** were highly selective for BuChE. We decided to study the effects of compounds **4f**, **4j**, **4k**, **4l**, **4m**, **4r**, **5a**, **7b**, and **7d** on the aluminium trichloride-induced behavioural inhibition model of zebrafish larvae. The result showed that compounds **4f**, **4j**, **4m**, and **7d** could alleviate the behavioural inhibition symptoms of zebrafish juveniles induced by aluminium trichloride. In particular, compound **4m** at 50.0 and 100.0 μg/mL concentration has significant effects compared with the model group. In conclusion, compound **4m** represents an interesting lead for drug design for the development of therapeutic or preventive agents for AD.

## 4. Experimental

### 4.1. Synthesis

Melting points were determined using a Thiele tube and were uncorrected. The ^1^H-NMR and ^13 ^C-NMR spectra were recorded with a Bruker AM-600 spectrometer (Billerica, MA) with TMS as the internal standard. Chemical shifts were reported at room temperature on a scale (ppm) with DMSO-d_6_ as the solvents and J values are given in Hertz. Mass spectra were obtained with an Agilent Trap VL LC/MS spectrometer (Santa Clara, CA). The absorbance was recorded by a Hitachi U-3000 UV spectrophotometer (Tokyo, Japan). Unless otherwise noted, all solvents and reagents were commercially available and used without further purification.

#### 4.1.1. General method for synthesis of compounds 3a–3c

Compound **1a** (1.38 g, 10 mmol) and P-aminophenylacetic acid (1.51 g, 10 mmol) were added to triethylamine (5.56 g, 55 mmol) and acetic anhydride (6.12 g, 60 mmol) and then the mixture was heated at 115 °C for 6 h. The resulting mixture was quenched by the addition of water, and a large amount of solid precipitated. The filtrate was vacuum filtered and the resulting solid was washed three times with water. The obtained solid **2a** was dissolved in 10 mL of absolute ethanol, and then reacted by adding hydrochloric acid (40 mL, 10%) at 80 °C for 6 h (TLC monitoring). The reaction solution was poured into 50 mL of ice water and stirred to precipitate a large amount of light green solid. After standing and filtration, the solid was washed with water until the pH of the wash solution was nearly neutral. The solid was recrystallised from ethanol/water to obtain compound **3a** (1.78 g, 70.4% yield). Compounds **3b–3c** were obtained using the same procedures.

#### 4.1.2. General method for synthesis of compounds 4a–4s

The 3-fluorobenzoyl chloride was synthesised from 3-fluorobenzoic acid (0.28 g, 2 mmol) with excess oxalyl chloride (1.28 mL, 15 mmol) in CH_2_Cl_2_ (20 mL) containing N, N-dimethylformamide (DMF) as a catalyst. The mixture was refluxed for about 2 h until the disappearance of the 3-fluorobenzoic acid monitoring by TLC, and then, the mixture was cooled to room temperature. The redundant oxalyl chloride was evaporated under vacuum. The crude benzoyl chloride was used in the following reaction without further purification. The 3-fluorobenzoyl chloride was dissolved in acetone (5 mL). Compound **3a** (0.51 g, 2 mmol) was added to 20 mL of acetone, and then 15 mL of pyridine was added, and 3-fluorobenzoyl chloride was added dropwise at 0–5 °C. The reaction was carried out for 7 h. The reaction solution was poured into ice water to precipitate a solid which was vacuum filtered, washed with water 3 times, and the solid was dried. The solid was added to ethanol for reflux washing, allowed to stand at cold, then vacuum filtered and rinsed with cold ethanol to give **4a** as a white solid (66% yield). Compounds **4 b–4s** were obtained using the same procedure.

##### 4.1.2.1. 3-[4-(3-fluoro-benzoylamino)-phenyl]-7-hydroxy-coumarin (4a)

White solid, yield: 66%, mp308.2–309.3 °C. ^1^H NMR (600 MHz, DMSO*-*d_6_) δ 10.59 (s, 1H), 10.43 (s, 1H), 8.18 (s, 1H), 7.89–7.82 (m, 3H), 7.83–7.77 (m, 1H), 7.76–7.70 (m, 2H), 7.61 (q, *J* = 6.7 Hz, 2H), 7.51–7.41 (m, 1H), 6.83 (dd, *J* = 8.5, 2.3 Hz, 1H), 6.77 (d, *J* = 2.2 Hz, 1H). ^13 ^C NMR (151 MHz, DMSO*-*d_6_) δ 164.64, 163.22, 161.60, 160.59, 155.27, 140.83, 139.21, 137.61, 137.57, 131.12, 131.07, 130.94, 130.37, 129.00, 124.42, 124.40, 122.14, 120.39, 119.11, 118.97, 115.08, 114.93, 113.88, 112.56, 102.19.MS: *m/z* (%):397.8 [M + 23] ^+^, 357.8.

##### 4.1.2.2. 3-[4-(4-fluoro-benzoylamino)-phenyl]-7-hydroxy-coumarin (4b)

White solid, yield: 70%, mp321.4–323.1 °C. ^1^H NMR (600 MHz, DMSO*-*d_6_) δ 10.59 (s, 1H), 10.38 (s, 1H), 8.17 (s, 1H), 8.12–8.03 (m, 2H), 7.88–7.82 (m, 2H), 7.76–7.70 (m, 2H), 7.61 (d, *J* = 8.5 Hz, 1H), 7.44–7.34 (m, 2H), 6.83 (dd, *J* = 8.5, 2.3 Hz, 1H), 6.77 (d, *J* = 2.2 Hz, 1H).^13^C NMR (151 MHz, DMSO*-*d_6_) δ 164.93, 163.77, 161.57, 160.60, 155.25, 140.77, 139.42, 131.74, 131.72, 130.96, 130.90, 130.76, 130.35, 128.98, 122.18, 120.33, 115.91, 115.77, 113.87, 112.56, 102.18. MS: *m/z* (%):375.9 [M + 1] ^+^, 279.7, 357.8.

##### 4.1.2.3. 3-[4-(4-methyl-benzoylamino)-phenyl]-7-hydroxy-coumarin (4c)

Light green solid, yield: 58%, mp307.5–308.9 °C. ^1^H NMR (600 MHz, DMSO*-*d_6_) δ 10.59 (s, 1H), 10.28 (s, 1H), 8.17 (s, 1H), 7.90 (d, *J* = 8.1 Hz, 2H), 7.86 (d, *J* = 8.7 Hz, 2H), 7.74–7.67 (m, 2H), 7.61 (d, *J* = 8.5 Hz, 1H), 7.35 (d, *J* = 8.0 Hz, 2H), 6.83 (dd, *J* = 8.5, 2.3 Hz, 1H), 6.76 (d, *J* = 2.1 Hz, 1H), 2.40 (s, 3H).^13^C NMR (151 MHz, DMSO*-*d_6_) δ 167.82, 161.58, 160.62, 155.27, 140.75, 139.65, 135.11, 133.65, 130.76, 130.69, 130.36, 130.14, 129.11, 128.85, 127.54, 126.88, 126.03, 125.58, 125.53, 122.24, 119.83, 113.88, 112.57, 102.20. MS: *m/z* (%):372.1 [M + 1] ^+^, 251.9, 279.9.

##### 4.1.2.4. 3-[4-(4-chloromethyl-benzoylamino)-phenyl]-7-hydroxy-coumarin (4d)

Light yellow, yield: 77%, mp264.9–267.0 °C. ^1^H NMR (600 MHz, DMSO*-*d_6_) δ 10.60 (s, 1H), 10.40 (s, 1H), 8.17 (s, 1H), 7.99 (d, *J* = 8.1 Hz, 2H), 7.86 (d, *J* = 8.6 Hz, 2H), 7.73 (d, *J* = 8.5 Hz, 2H), 7.61 (d, *J* = 8.3 Hz, 3H), 6.83 (dd, *J* = 8.4, 1.7 Hz, 1H), 6.77 (d, *J* = 1.3 Hz, 1H), 4.86 (s, 2H).^13^C NMR (151 MHz, DMSO*-*d_6_) δ 165.60, 161.58, 160.60, 155.25, 141.62, 140.77, 139.44, 135.15, 130.75, 130.35, 129.28, 128.98, 128.57, 122.18, 120.30, 113.87, 112.56, 102.18. MS: *m/z* (%):406.0 [M + 1] ^+^, 351.8, 369.9, 387.9.

##### 4.1.2.5. 3-[4-(3-methyl-benzoylamino)-phenyl]-7-hydroxy-coumarin (4e)

Light yellow solid, yield: 85%, mp265.2–266.7 °C. ^1^H NMR (600 MHz, DMSO*-*d_6_) δ 10.59 (s, 1H), 10.33 (s, 1H), 8.17 (s, 1H), 7.86 (d, *J* = 8.7 Hz, 2H), 7.80 (s, 1H), 7.77 (d, *J*= 6.5 Hz, 1H), 7.72 (d, *J*= 8.7 Hz, 2H), 7.61 (d, *J* = 8.5 Hz, 1H), 7.43 (d, *J* = 6.6 Hz, 2H), 6.83 (dd, *J* = 8.5, 2.2 Hz, 1H), 6.76 (d, *J* = 2.2 Hz, 1H), 2.42 (s, 3H).^13^CNMR (151 MHz, DMSO*-*d_6_) δ 166.16, 161.56, 160.61, 155.24, 140.72, 139.57, 138.20, 135.32, 132.70,130.62, 130.34, 128.96, 128.79, 128.63, 125.34, 122.21, 120.26, 113.86, 112.57, 102.18, 56.50. MS: *m/z* (%):371.9 [M + 1] ^+^, 251.8, 279.8.

##### 4.1.2.6. 3-[4-(3-chloro-benzoylamino)-phenyl]-7-hydroxy-coumarin (4f)

Yellow solid, yield: 81%, mp304.9–306.0 °C.1H NMR (600 MHz, DMSO*-*d_6_) δ 10.60 (s, 1H), 10.47 (s, 1H), 8.18 (s, 1H), 8.04 (t, *J* = 1.8 Hz, 1H), 7.98–7.90 (m, 1H), 7.88–7.79 (m, 2H), 7.77–7.71 (m, 2H), 7.68 (ddd, *J* = 8.0, 2.1, 1.0 Hz, 1H), 7.60 (dd, *J* = 15.8, 8.1 Hz, 2H), 6.83 (dd, *J* = 8.5, 2.3 Hz, 1H), 6.77 (d, *J* = 2.2 Hz, 1H).^13^C NMR (150 MHz, DMSO*-*d_6_) δ 164.55, 161.60, 160.59, 155.27, 140.83, 139.21, 137.26, 133.71, 131.96, 130.96, 130.92, 130.37, 129.01, 127.92, 127.02, 122.14, 120.39, 113.88, 112.56, 102.19. MS: *m/z* (%):413.9 [M + 23]^+^, 373.8.

##### 4.1.2.7. 3-[4-(2-fluoro-benzoylamino)-phenyl]-7-hydroxy-coumarin (4g)

Light red, solid yield: 72%, mp268.9–372.4 °C.1H NMR (600 MHz, DMSO*-*d_6_) δ 10.59 (s, 1H), 10.54 (s, 1H), 8.16 (s, 1H), 7.80 (d, *J* = 8.7 Hz, 2H), 7.71 (dd, *J* = 17.1, 5.1 Hz, 3H), 7.61 (d, *J* = 8.5 Hz, 2H), 7.36 (dd, *J* = 10.0, 2.5 Hz, 2H), 6.83 (dd, *J* = 8.5, 2.3 Hz, 1H), 6.77 (d, *J* = 2.2 Hz, 1H).^13^C NMR (151 MHz, DMSO*-*d_6_) δ 163.29, 161.59, 160.59, 155.27, 140.80, 139.17, 130.90, 130.42, 130.40, 130.36, 129.13, 125.07, 125.05, 122.14, 119.73, 116.73, 116.59, 113.87, 112.55, 102.18. MS: *m/z* (%):376.0 [M + 1]^+^, 279.8, 357.9.

##### 4.1.2.8. 3-[4-(4-chloro-benzoylamino)-phenyl]-7-hydroxy-coumarin (4h)

Light green solid, yield: 80%, mp313.2–315.0 °C.1H NMR (600 MHz, DMSO*-*d_6_) δ 10.59 (s, 1H), 10.43 (s, 1H), 8.17 (s, 1H), 8.08–7.98 (m, 2H), 7.89–7.82 (m, 2H), 7.78–7.69 (m, 2H), 7.67–7.57 (m, 3H), 6.83 (dd, *J* = 8.5, 2.3 Hz, 1H), 6.76 (d, *J* = 2.2 Hz, 1H).^13^C NMR (151 MHz, DMSO*-*d_6_) δ 164.93, 161.58, 160.59, 155.26, 140.80, 139.31, 136.96, 133.99, 130.86, 130.36, 130.15, 128.99, 128.97, 122.16, 120.36, 113.87, 112.56, 102.18. MS: *m/z* (%):391.9 [M + 1]^+^, 279.8, 373.8.

##### 4.1.2.9. 3-[4-(3, 5-dimethyl-benzoylamino)-phenyl]-7-hydroxy-coumarin (4i)

White solid, yield: 72%, mp250.2–252.3 °C.1H NMR (600 MHz, DMSO*-*d_6_) δ 10.58 (s, 1H), 10.28 (s, 1H), 8.17 (s, 1H), 7.85 (d, *J* = 8.7 Hz, 2H), 7.75–7.66 (m, 2H), 7.64–7.53 (m, 3H), 7.23 (s, 1H), 6.83 (dd, *J* = 8.5, 2.2 Hz, 1H), 6.76 (d, *J* = 2.2 Hz, 1H), 4.35 (t, *J* = 5.1 Hz, 1H), 2.37 (s, 6H).^13^C NMR (151 MHz, DMSO*-*d_6_) δ 166.27, 161.55, 160.61, 155.24, 140.70, 139.61, 138.05, 135.35, 133.39, 130.56, 130.34, 128.94, 125.86, 122.23, 120.23, 113.86, 112.57, 102.18, 56.50. MS: *m/z* (%):386.1 [M + 1]^+^, 251.9, 279.9.

##### 4.1.2.10. 3-[4-(3-fluoro-benzoylamino)-phenyl]-8-methoxy-coumarin (4j)

White solid, yield: 75%, mp259.8–262.4 °C.1H NMR (600 MHz, DMSO*-*d_6_) δ 10.47 (s, 1H), 8.27 (s, 1H), 7.91–7.86 (m, 2H), 7.85 (d, *J* = 7.9 Hz, 1H), 7.80 (ddd, *J* = 11.3, 6.3, 2.1 Hz, 3H), 7.61 (td, *J* = 8.0, 5.9 Hz, 1H), 7.48 (dd, *J* = 8.5, 2.3 Hz, 1H), 7.38–7.27 (m, 3H), 3.94 (s, 3H).^13^C NMR (151 MHz, DMSO*-*d_6_) δ 164.70, 161.60, 159.96, 146.72, 142.61, 140.40, 139.78, 137.56, 131.13, 131.08, 130.34, 129.32, 126.88, 125.02, 124.44, 124.42, 120.61, 120.36, 120.23, 115.10, 114.95, 114.25, 56.57. MS: *m/z* (%):390.1 [M + 1]^+^, 293.9, 372.0.

##### 4.1.2.11. 3-[4-(4-fluoro-benzoylamino)-phenyl]-8-methoxy-coumarin (4k)

White solid, yield: 78%, mp296.5–297.8 °C.1H NMR (600 MHz, DMSO*-*d_6_) δ 10.42 (s, 1H), 8.26 (s, 1H), 8.07 (s, 2H), 7.88 (d, *J* = 8.1 Hz, 2H), 7.78 (d, *J* = 8.2 Hz, 2H), 7.39 (t, *J* = 7.6 Hz, 2H), 7.36–7.26 (m, 3H), 3.94 (s, 3H).^13^C NMR (151 MHz, DMSO*-*d_6_) δ 165.00, 163.79, 159.97, 146.72, 142.60, 140.34, 139.99, 131.70, 130.99, 130.93, 130.16, 129.30, 126.91, 125.02, 120.62, 120.30, 120.23, 115.93, 115.79, 114.24, 56.58. MS: *m/z* (%):390.1 [M + 1]^+^, 293.9, 371.9.

##### 4.1.2.12. 3-[4-(4-trifluoromethyl-benzoylamino)-phenyl]-8-methoxy-coumarin (4l)

White solid, yield: 68%, mp298.1–301.5 °C.1H NMR (600 MHz, DMSO*-*d_6_) δ 10.63 (s, 1H), 8.27 (s, 1H), 8.18 (d, *J* = 8.1 Hz, 2H), 7.94 (d, *J* = 8.3 Hz, 2H), 7.90 (d, *J* = 8.8 Hz, 2H), 7.84–7.76 (m, 2H), 7.40–7.27 (m, 3H), 3.94 (s, 3H).^13^C NMR (151 MHz, DMSO*-*d_6_) δ 164.95, 159.95, 146.72, 142.62, 140.44, 139.71, 130.47, 129.36, 129.15, 126.86, 125.92, 125.89, 125.02, 120.61, 120.38, 120.24, 114.26, 56.57. MS: *m/z* (%):461.9 [M + 23]^+^, 419.9.

##### 4.1.2.13. 3-[4-(4-chloromethyl-benzoylamino)-phenyl]-8-methoxy-coumarin (4m)

Yellow solid, yield: 70%, mp274.2–275.6 °C.1H NMR (600 MHz, DMSO*-*d_6_) δ 10.43 (s, 1H), 8.26 (s, 1H), 8.03–7.96 (m, 2H), 7.91–7.86 (m, 2H), 7.81–7.76 (m, 2H), 7.61 (d, *J* = 8.3 Hz, 2H), 7.37–7.29 (m, 3H), 4.86 (s, 2H), 3.94 (s, 3H).^13^C NMR (151 MHz, DMSO*-*d_6_) δ 165.66, 159.96, 146.72, 142.60, 141.66, 140.33, 140.01, 135.10, 130.15, 129.30, 128.59, 126.91, 125.02, 120.62, 120.27, 120.23, 114.23, 56.57. MS: *m/z* (%):419.9 [M + 1]^+^, 383.3.

##### 4.1.2.14. 3-[4-(3-chloro-benzoylamino)-phenyl]-8-methoxy-coumarin (4n)

White solid, yield: 81%, mp265.3–266.7 °C.1H NMR (600 MHz, DMSO*-*d_6_) δ 10.50 (s, 1H), 8.27 (s, 1H), 8.04 (s, 1H), 7.95 (d, *J* = 7.5 Hz, 1H), 7.88 (d, *J* = 8.2 Hz, 2H), 7.79 (d, *J* = 8.2 Hz, 2H), 7.69 (d, *J* = 7.8 Hz, 1H), 7.59 (t, *J* = 7.8 Hz, 1H), 7.41–7.24 (m, 3H), 3.94 (s, 3H).^13^C NMR (151 MHz, DMSO*-*d_6_) δ 164.63, 159.96, 146.73, 142.62, 140.41, 139.78, 137.22, 133.72, 132.01, 130.94, 130.36, 129.33, 129.31, 127.94, 127.05, 126.88, 125.03, 120.62, 120.36, 120.24, 114.27, 56.58. MS: *m/z* (%):428.0 [M + 23]^+^, 265.9, 293.9.

##### 4.1.2.15. 3-[4-(3-methyl-benzoylamino)-phenyl]-8-methoxy-coumarin (4o)

Light yellow solid, yield: 87%, mp244.4–247.0 °C.1H NMR (600 MHz, DMSO*-*d_6_) δ 10.36 (s, 1H), 8.26 (s, 1H), 7.93–7.85 (m, 2H), 7.78 (dd, *J* = 14.6, 7.8 Hz, 4H), 7.48–7.39 (m, 2H), 7.36–7.27 (m, 3H), 3.94 (s, 3H), 2.42 (s, 3H).^13^C NMR (151 MHz, DMSO*-*d_6_) δ 166.23, 159.97, 146.72, 142.59, 140.28, 140.14, 138.21, 135.28, 132.74, 130.02, 129.27, 128.80, 128.65, 126.93, 125.36, 125.01, 120.63, 120.23, 114.21, 56.57. MS: *m/z* (%):386.2 [M + 1]^+^, 265.9, 293.9.

##### 4.1.2.16. 3-[4-(2-fluoro-benzoylamino)-phenyl]-8-methoxy-coumarin (4p)

White solid, yield: 82%, mp243.1–245.2 °C.1H NMR (600 MHz, DMSO*-*d_6_) δ 10.58 (s, 1H), 8.25 (s, 1H), 7.83 (d, *J* = 8.7 Hz, 2H), 7.78 (d, *J* = 8.7 Hz, 2H), 7.70 (d, *J* = 1.5 Hz, 1H), 7.60 (d, *J* = 8.1 Hz, 1H), 7.44–7.24 (m, 5H), 3.94 (s, 3H).^13^C NMR (151 MHz, DMSO*-*d_6_) δ 163.37, 159.95, 146.72, 142.61, 140.38, 139.72, 133.14, 130.43, 130.41, 130.32, 129.45, 126.87, 125.41, 125.31, 125.09, 125.07, 125.02, 120.60, 120.24, 119.71, 116.75, 116.60, 114.26, 56.57. MS: *m/z* (%):411.9 [M + 23]^+^, 293.9, 371.9.

##### 4.1.2.17. 3-[4-(2-methoxy-benzoylamino)-phenyl]-8-methoxy-coumarin (4q)

Light yellow solid, yield: 77%, mp205.9–206.3 °C.1H NMR (600 MHz, DMSO*-*d_6_) δ 10.27 (s, 1H), 8.24 (s, 1H), 7.84 (d, *J* = 8.7 Hz, 2H), 7.76 (d, *J* = 8.7 Hz, 2H), 7.66 (dd, *J* = 7.5, 1.6 Hz, 1H), 7.58–7.48 (m, 1H), 7.34 (dd, *J* = 6.6, 4.1 Hz, 1H), 7.31 (dd, *J* = 4.6, 3.0 Hz, 2H), 7.20 (d, *J* = 8.3 Hz, 1H), 7.08 (td, *J* = 7.5, 0.8 Hz, 1H), 3.94 (s, 3H), 3.92 (s, 3H).^13^C NMR (151 MHz, DMSO*-*d_6_) δ 165.13, 159.97, 156.98, 146.71, 142.59, 140.23, 139.94, 132.61, 130.16, 129.94, 129.38, 126.92, 125.34, 125.00, 120.99, 120.62, 120.22, 119.64, 114.21, 112.50, 56.57. MS: *m/z* (%):462.0 [M + 1]^+^, 134.9.

##### 4.1.2.18. 3-[4-(2, 3-dichloro-benzoylamino)-phenyl]-8-methoxy-coumarin (4r)

White solid, yield: 62%, mp258.6–261.7 °C.1H NMR (600 MHz, DMSO*-*d_6_) δ 10.76 (s, 1H), 8.25 (s, 1H), 7.83–7.74 (m, 5H), 7.61 (dd, *J* = 7.6, 1.5 Hz, 1H), 7.51 (t, *J* = 7.8 Hz, 1H), 7.37–7.28 (m, 3H), 3.94 (s, 3H).^13^C NMR (151 MHz, DMSO*-*d_6_) δ 164.72, 159.95, 146.73, 142.63, 140.44, 139.54, 132.58, 131.91, 130.51, 129.54, 129.22, 128.62, 127.90, 126.86, 125.03, 120.59, 120.25, 119.59, 114.29, 56.58. MS: *m/z* (%):391.9 [M + 1]^+^, 293.9, 421.9.

##### 4.1.2.19. 3-[4-(4-Chloromethyl-benzoylamino)-phenyl]-7, 8-dihydroxy-coumarin (4s)

Yellow solid, yield: 58%, mp285.3–286.7 °C.1H NMR (600 MHz, DMSO*-*d_6_) δ 10.40 (s, 1H), 10.13 (s, 1H), 9.41 (s, 1H), 8.13 (s, 1H), 7.99 (d, *J* = 8.2 Hz, 2H), 7.86 (d, *J* = 8.7 Hz, 2H), 7.74 (d, *J* = 8.7 Hz, 2H), 7.61 (d, *J* = 8.3 Hz, 2H), 7.11 (d, *J* = 8.5 Hz, 1H), 6.84 (d, *J* = 8.4 Hz, 1H), 4.86 (s, 2H).^13^C NMR (151 MHz, DMSO*-*d_6_) δ 165.59, 160.57, 149.94, 143.54, 141.61, 141.34, 139.39, 135.15, 132.28, 130.85, 129.28, 129.01, 128.57, 121.92, 120.29, 119.55, 113.35, 113.28. MS: *m/z* (%):422.0 [M + 1]^+^, 368.0, 385.9, 403.9.

#### 4.1.3. General method for synthesis of compounds 5a–5b, 6a–6b, and 7a–7d

HOBt (2 mmol) and dicyclohexyl carbodiimide (DCC, 2 mmol) were added into an anhydrous toluene solution (10 mL) containing compounds **1–6** (2 mmol) at 0 °C, and the mixture was stirred for 45 min. Then, compounds **3a–3b** (2.2 mmol) were added to the reaction solution, respectively. There action mixture was stirred at room temperature for 8–16 h until compounds **1–6** disappeared. The reaction mixture was filtered and the toluene was evaporated under reduced pressure. The residue was dissolved in 10% NaOH and filtered, and then 15% HCl was added to adjust pH of the solution to 3–4, followed by the production of precipitation. The precipitation washed with water 3 times, and the solid was dried. The solid was added to ethanol for reflux washing, allowed to stand at cold, then vacuum filtered and rinsed with cold ethanol to give compounds **5a–5b**, **6a–6b**, and **7a–7d**.

##### 4.1.3.1. 3-[4-(furan-2-formylamino)-phenyl]-7-hydroxy-coumarin (5a)

Light yellow solid, yield: 70%, mp242.6**–**244.3 °C.1H NMR (600 MHz, DMSO*-*d_6_) δ 10.59 (s, 1H), 10.30 (s, 1H), 8.18–8.15 (m, 1H), 7.96 (dd, *J* = 5.6, 0.9 Hz, 1H), 7.83 (d, *J* = 8.7 Hz, 2H), 7.71 (d, *J* = 8.7 Hz, 2H), 7.60 (d, *J* = 8.5 Hz, 1H), 7.38**–**7.36 (m, 1H), 6.83 (dd, *J* = 8.5, 2.2 Hz, 1H), 6.76 (d, *J* = 2.1 Hz, 1H), 6.74**–**6.70 (m, 1H).^13^C NMR (150 MHz, DMSO*-*d_6_) δ 161.57, 160.59, 156.69, 155.25, 147.92, 146.30, 140.78, 138.89, 130.76, 130.35, 129.00, 122.18, 120.31, 115.37, 113.87, 112.69, 112.56, 102.18. MS: *m/z* (%):347.9 [M + 1]^+^, 279.8, 347.8.

##### 4.1.3.2. 3-[4-(furan-2-formylamino)-phenyl]-8-methoxy-coumarin (5b)

Yellow solid, yield: 75%, mp268.1**–**269.5 °C.1H NMR (600 MHz, DMSO*-*d_6_) δ 10.34 (s, 1H), 8.25 (s, 1H), 8.00**–**7.94 (m, 1H), 7.91**–**7.83 (m, 2H), 7.80**–**7.72 (m, 2H), 7.39**–**7.37 (m, 1H), 7.35**–**7.29 (m, 3H), 6.73 (dd, *J* = 3.5, 1.7 Hz, 1H), 3.94 (d, *J* = 5.1 Hz, 3H).^13^C NMR (151 MHz, DMSO*-*d_6_) δ 159.96, 156.73, 147.88, 146.72, 146.36, 142.60, 140.34, 139.47, 130.16, 129.31, 126.90, 125.02, 120.61, 120.27, 120.22, 115.46, 114.24, 112.70. MS: *m/z* (%):362.0 [M + 1]^+^, 298.0.

##### 4.1.3.3. 3-[4-(1-naphthoylamino)-phenyl]-7-hydroxy-coumarin (6a)

Light green solid, yield: 78%, mp287.5**–**289.3 °C.1H NMR (600 MHz, DMSO*-*d_6_) δ 10.70 (s, 1H), 10.60 (s, 1H), 8.21 (dd, *J* = 7.0, 2.5 Hz, 1H), 8.18 (s, 1H), 8.10 (d, *J* = 8.3 Hz, 1H), 8.06**–**8.00 (m, 1H), 7.89 (d, *J* = 8.6 Hz, 2H), 7.79 (d, *J* = 6.3 Hz, 1H), 7.75 (d, *J* = 8.7 Hz, 2H), 7.65**–**7.58 (m, 4H), 6.84 (dd, *J* = 8.4, 2.3 Hz, 1H), 6.77 (d, *J* = 2.2 Hz, 1H).^13^C NMR (151 MHz, DMSO*-*d_6_) δ 167.82, 161.58, 160.62, 155.27, 140.75, 139.65, 135.11, 133.65, 130.76, 130.69, 130.36, 130.14, 129.11, 128.85, 127.54, 126.88, 126.03, 125.58, 125.53, 122.24, 119.83, 113.88, 112.57, 102.20. MS: *m/z* (%):408.1 [M + 1]^+^, 251.9, 279.9.

##### 4.1.3.4. 3-[4-(1-naphthoylamino)-phenyl]-8-methoxy-coumarin (6b)

White solid, yield: 82%, mp275.4**–**277.3 °C.1H NMR (600 MHz, DMSO*-*d_6_) δ 10.75 (s, 1H), 8.26 (s, 1H), 8.25**–**8.18 (m, 1H), 8.10 (d, *J* = 8.3 Hz, 1H), 8.07**–**7.99 (m, 1H), 7.92 (d, *J* = 8.6 Hz, 2H), 7.84**–**7.74 (m, 3H), 7.68**–**7.55 (m, 3H), 7.40**–**7.25 (m, 3H), 3.94 (s, 3H).^13^C NMR (151 MHz, DMSO*-*d_6_) δ 167.89, 159.98, 146.73, 142.62, 140.32, 140.20, 135.05, 133.65, 130.73, 130.17, 130.13, 129.43, 128.85, 127.56, 126.97, 126.89, 126.07, 125.57, 125.53, 125.03, 120.63, 120.24, 119.81, 114.25, 56.58. MS: *m/z* (%):422.2 [M + 1]^+^, 155.0, 266.0, 294.0.

##### 4.1.3.5. 3-[4-(2-naphthoylamino)-phenyl]-7-hydroxy-coumarin (7a)

Light yellow solid, yield: 65%, mp305.6**–**307.2 °C.1H NMR (600 MHz, DMSO*-*d_6_) δ 10.60 (s, 1H), 10.56 (s, 1H), 8.62 (s, 1H), 8.19 (s, 1H), 8.13**–**8.10 (m, 1H), 8.09–8.01 (m, 3H), 7.92 (d, *J* = 8.7 Hz, 2H), 7.78**–**7.72 (m, 2H), 7.64 (ddd, *J* = 17.6, 11.8, 5.0 Hz, 3H), 6.84 (dd, *J* = 8.4, 2.3 Hz, 1H), 6.77 (d, *J* = 2.2 Hz, 1H).^13^C NMR (151 MHz, DMSO*-d*_6_) δ 166.10, 161.57, 160.62, 155.26, 140.76, 139.60, 134.79, 132.62, 132.56, 130.71, 130.36, 129.46, 129.01, 128.53, 128.36, 128.17, 127.37, 124.94, 122.21, 120.32, 113.88, 112.58, 102.19, 56.50. MS: *m/z* (%):408.1 [M + 1]^+^, 251.9, 279.9.

##### 4.1.3.6. 3-[4-(2-naphthoylamino)-phenyl]-8-methoxy-coumarin (7b)

Yellow solid, yield: 72%, mp242.1**–**243.5 °C.1H NMR (600 MHz, DMSO*-*d_6_) δ 10.60 (s, 1H), 8.63 (s, 1H), 8.28 (s, 1H), 8.07 (ddd, *J* = 24.6, 15.8, 7.5 Hz, 4H), 7.98**–**7.93 (m, 2H), 7.85**–**7.78 (m, 2H), 7.65 (td, *J* = 7.4, 1.4 Hz, 2H), 7.33 (ddd, *J* = 7.8, 4.7, 2.6 Hz, 3H), 3.94 (s, 3H).^13^C NMR (151 MHz, DMSO*-*d_6_) δ 166.17, 159.98, 146.73, 142.60, 140.32, 140.17, 134.81, 132.58, 132.56, 130.11, 129.47, 129.33, 128.57, 128.54, 128.38, 128.18, 127.38, 126.93, 125.02, 124.94, 120.64, 120.29, 120.23, 114.23, 56.57. MS: *m/z* (%):422.1 [M + 1]^+^, 265.9, 293.9.

##### 4.1.3.7. 3-[4-(6-chloro-N-nicotinamide)-phenyl]-8-methoxy-coumarin (7c)

Light yellow solid, yield:65%, mp310.9**–**313.2 °C.1H NMR (600 MHz, DMSO*-*d_6_) δ 10.64 (s, 1H), 8.98 (d, *J*= 2.3 Hz, 1H), 8.39 (dd, *J* = 8.3, 2.5 Hz, 1H), 8.27 (s, 1H), 7.87 (d, *J* = 8.7 Hz, 2H), 7.82**–**7.78 (m, 2H), 7.73 (d, *J* = 8.3 Hz, 1H), 7.36**–**7.29 (m, 3H), 3.94 (d, *J* = 4.7 Hz, 3H).^13^C NMR (151 MHz, DMSO*-d*_6_) δ 163.50, 149.88, 149.86, 146.73, 140.50, 139.61, 129.41, 129.38, 125.05, 124.65, 120.61, 120.36, 120.33, 120.26, 114.30, 56.58. MS: *m/z* (%):429.0 [M + 23]^+^, 265.9, 293.9.

##### 4.1.3.8. 3-[4-(thiophene-2-formylamino)-phenyl]-8-methoxy-coumarin (7d)

Yellow solid, yield: 72%, mp263.5**–**265.4 °C.1H NMR (600 MHz, DMSO*-*d_6_) δ 10.38 (s, 1H), 8.26 (s, 1H), 8.07 (dd, *J* = 3.8, 0.9 Hz, 1H), 7.89 (dd, *J* = 5.0, 0.9 Hz, 1H), 7.85 (d, *J* = 1.7 Hz, 1H), 7.84 (d, *J* = 1.8 Hz, 1H), 7.79 (d, *J* = 1.8 Hz, 1H), 7.77 (d, *J* = 1.8 Hz, 1H), 7.37**–**7.28 (m, 3H), 7.25 (dd, *J* = 4.9, 3.8 Hz, 1H), 3.94 (s, 3H).^13^C NMR (151 MHz, DMSO*-*d_6_) δ 160.44, 159.95, 146.72, 142.60, 140.37, 140.35, 139.65, 132.61, 130.15, 129.80, 129.34, 128.61, 126.86, 125.02, 120.62, 120.25, 120.23, 114.23, 56.57. MS: *m/z* (%):399.9 [M + 23]^+^, 265.9, 293.9.

### 4.2. Biological activity

#### 4.2.1. Preparation of main reagents

In the experiment, 0.05 mol/L phosphate buffered saline (PBS) of pH 8.0 was prepared first, and then the following solutions were prepared: 1.5 mmol/L Acetylthiocholine iodide (ATChI); 0.75 mmol/L 5, 5'-Dithiobis-(2-nitrobenzoic acid) (DTNB, Sigma Aldrich, St. Louis, MO); 0.2 U/mL Acetylcholinesterase (AChE, from electric squid, Macklin); 0.5 U/mL Butyrylcholinesterase (BuChE, from equine serum, Aladdin, Shanghai, China); 1.5 mmol/L S-Butyrylthiocholine iodide (BTChI, Sigma Aldrich, St. Louis, MO). 4% sodium dodecyl sulphate (SDS) was prepared with secondary water. The test compounds and the positive control Donepezil (Sigma Aldrich, St. Louis, MO) were formulated with DMSO to a gradient of 0.1, 1.0, 10.0, 50.0, 100.0, 500.0, and 1000.0 μg/mL. The above reagents were all analytically pure, and the test water was all distilled water. All solutions were stored in a refrigerator at 4 °C.

#### 4.2.2. In vitro AChE/BuChE inhibitory activity

The AChE/BuChE inhibitory activity of the 3–(4-aminophenyl)-coumarin derivatives was determined by the method of Ellaman^28^ with slight modifications. The experiment was divided into control group, sample blank group and sample group. Take a series of test tubes, the reagents were loaded in test tubes at the dose of Table 6. After incubation at 37 °C for 20 min, quickly add 1 mL of 4% SDS to stop the reaction. Measuring ultraviolet absorbance immediately at 412 nm. All experiments were run in triplicate. The absorbance was determined by UV spectrophotometer, and the inhibition rate of AChE and the IC_50_ value of each sample were calculated according to the formula.

$$\text{Inhibition rate }\%\;=\;\left\{[\left({\text{A}}_{\text{C}}-{\text{A}}_{\text{S}}\right)-{\text{A}}_{\text{SB}}]/{\text{A}}_{\text{C}}\right\}\;\times \text{1}00\%,$$

where A_C_ (no sample added) is the absorbance of control group; A_S_ is the absorbance of sample group; A_SB_ (add sample solution but no substrate ATChI added) is the absorbance of sample blank group.

**Table 6.** The amount and order of each reactant of acetylcholinesterase inhibition test.

Reagents	Volume (μL)		
	Control group	Sample blank group	Sample group
PBS	2650	2650	2650
AChE	50	50	50
DNTB	100	100	100
Compounds	0	100	100
DMSO	100	0	0
Mix well and incubate at 37 °C for 5 min			
ATChI	100	0	100
PBS	0	100	0
Mix well and react at 37 °C for 20 min			
SDS	1000	1000	1000

The method for determining BuChE is the same as the method for measuring AChE. The sample can be added with reference to Table 6, AChE is changed to BuChE, and the substrate ATChI is changed to substrate BTChI. The order of loading and the amount of loading are the same as Table 6. The absorbance was determined by UV spectrophotometer, and the inhibition rate of BuChE and the IC_50_ value of each sample were calculated according to the formula. The formula used is the same as AChE.

#### 4.2.3. Behavioural experiment of zebrafish juveniles

##### 4.2.3.1. Zebrafish juveniles

AB wild-type zebrafish is provided by the Key Laboratory of Drug Screening Technology of Shandong Academy of Sciences. The zebrafish culture was carried out in a light cycle of 14 h light and 10 h dark at a temperature of 28 °C. At 16:00 the day before the experiment, the healthy zebrafish were mated in a tank with a male to female ratio of 1:1 or 2:1. The next day at 8:30 to extract the separator, after 1–2 h, collect the fertilised eggs, wash the fertilised eggs with culture water for 3 times, disinfect the fertilised eggs with methylene blue solution, and then move into clean zebrafish culture water at about 28 °C culture in a light incubator (HPG-280BX).

##### 4.2.3.2. Model of exercise retardation in Zebrafish juveniles made of aluminium trichloride

The 72 hpf zebrafish were placed in a petri dish, and the normal developing juveniles were selected under a microscope in a 12-well plate. The concentration of AlCl_3_ in the experiment was set to 0, 0.1, 0.5, 1.0, 5.0, 10.0, and 50.0 mg/L (prepared for aquaculture water), three replicate wells were set in each concentration group and then placed in a constant temperature incubator at 28 °C to continue the culture. The continuous drug solution was exposed for 3 days. The different concentrations of AlCl_3_ and the blank control group were placed in 48-well plates. Each well was juvenile fish developed to 72 h post-exposure (hpe) and added 0.5 mL of culture water and 8 juveniles were set in each experimental group. The well plate was placed in the dark box of the zebrafish behaviour analysis system. The fish was first adapted to 10 min at the beginning of the experiment. The trajectories of the juveniles in each group were collected using zeblab software for 10 min, recorded every 5 min, and exported by software. Then calculate the total distance of each group of zebrafish juveniles. The effects of different concentrations of aluminium trichloride on the death and teratogenicity of zebrafish juveniles were observed by microscopy.

##### 4.2.3.3. Effects of compounds on the inhibition of movement ability of zebrafish caused by aluminium trichloride

The zebrafish with 72 hpf after fertilisation is placed in a petri dish. Under normal conditions, the normal developing juvenile fish are selected in a 12-well plate, and 15 juveniles per hole are set. Control group (0.5% DMSO), model group (5.0 mg/L AlCl_3_), 5.0 mg/L AlCl_3_ + test compound 5 μg/mL group, 5.0 mg/L AlCl_3_ + test compound 10 μg/mL group, 5.0 mg/L AlCl_3_ + test compound 50 μg/mL group, 5.0 mg/L AlCl_3_ + test compound 100 μg/mL group, three duplicate wells were set at each concentration. After the administration, the zebrafish was placed in a constant temperature incubator at 28 °C to continue the culture for 3 days. Changing the liquid (aquaculture water) every 24 h and removing dead young fish. Behavioural observations are consistent with AlCl_3_ modelling.

#### 4.2.4. Statistical analysis

The experimental data were processed by the statistical software SPSS version 16.0 (SPSS Inc., Chicago, IL). All the experimental data were expressed by mean ± SD or mean ± SE. The statistical differences were analysed by ANOVA. The comparison between groups was performed by Tukey test. *p* < .05 was considered as significant difference. *p* < .01 was a very significant difference.

## Supplementary Material

Supplemental Material
