# Supplementary material for: Synthesis and biological evaluation of 3–(4-aminophenyl)-coumarin derivatives as potential anti-Alzheimer’s disease agents
Source: J Enzyme Inhib Med Chem. 2019 May 22;34(1):1083–92. doi: 10.1080/14756366.2019.1615484 (PMC6534212; doi:10.1080/14756366.2019.1615484)

Synthesis and biological evaluation of 3-(4-aminophenyl)-coumarin derivatives as potential anti-Alzheimer's disease agents

Yuheng Hu [1,2,3,4,] †, Jie Yang [1,2,3,4,] †, Liying Wang [5], Teng Liu [1,2,3,4], Jie Sun[1,2,3,4,] * and Xiaojing Wang [1,2,3,4,] *

[1] School of Medicine and Life Sciences, University of Jinan-Shandong Academy of Medical Sciences, Jinan 250200, Shandong, China
[2] Institute of MateriaMedica, Shandong Academy of Medical Sciences, Jinan 250062, Shandong, China
[3] Key Laboratory for Biotech-Drugs Ministry of Health, Jinan 250062, Shandong, China
[4] Key Laboratory for Rare & Uncommon Diseases of Shandong Province, Jinan 250062, Shandong, China
[5] Shandong Electric Power Central Hospital, Jinan250001, Shandong, China
* Correspondence: sunjie310@126.com (J.S.); xiaojing6@gmail.com (X.J.W.).
† These authors contributed equally to this work.

^{1}H NMR(600 MHz, DMSO-d_6) and ^{13}C NMR(151 MHz, DMSO-d_6) of compounds **4a-4s**, **5a-5b**, **6a-6b**, **7a-7d**.

compound **4a** ^{1}HNMR

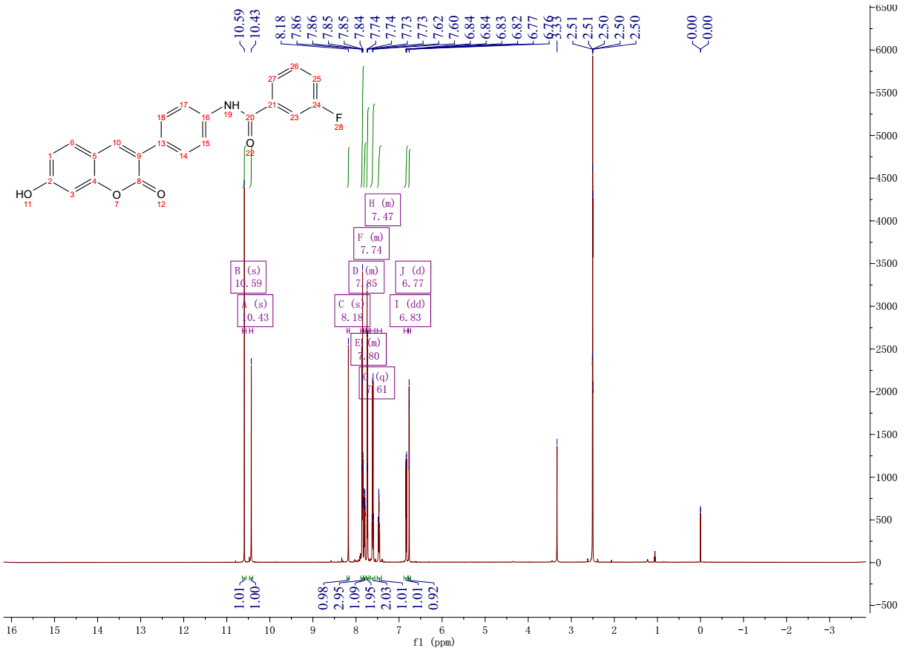

compound **4a** ^{13}CNMR

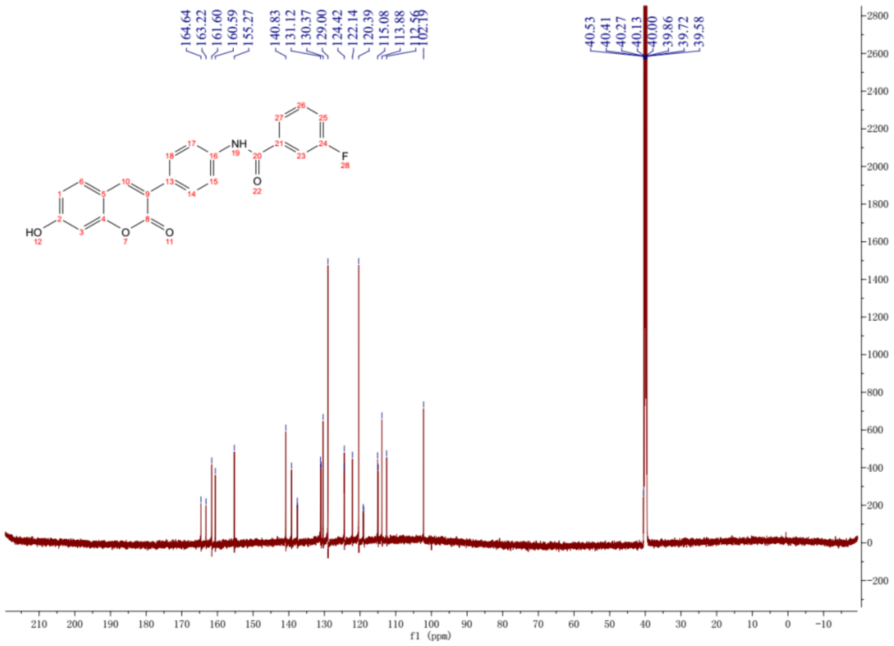

compound **4b** ^{1}HNMR

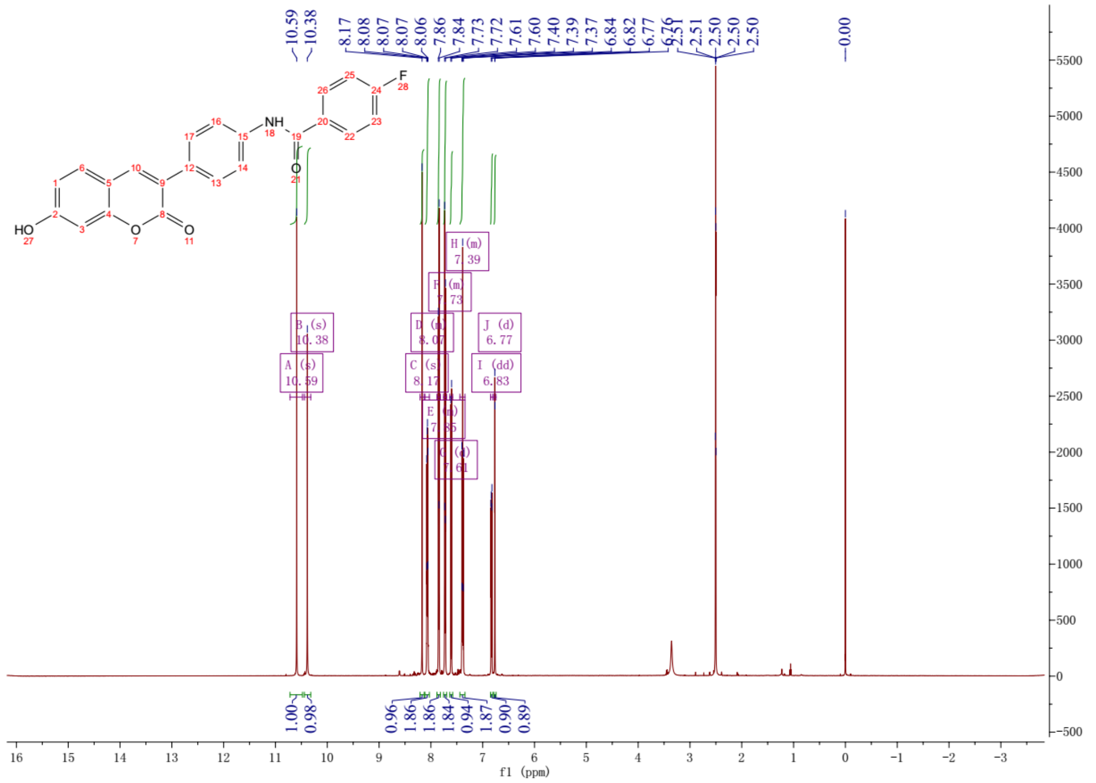

compound **4b** ^{13}CNMR

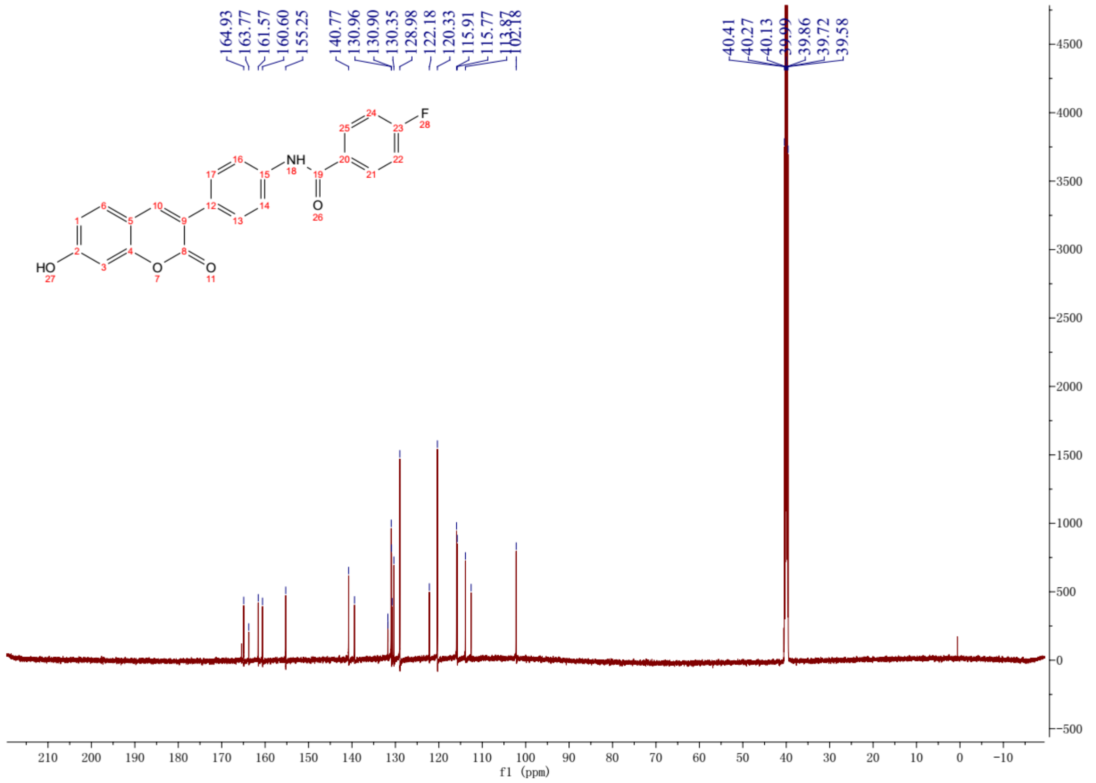

compound **4c** ^{1}HNMR

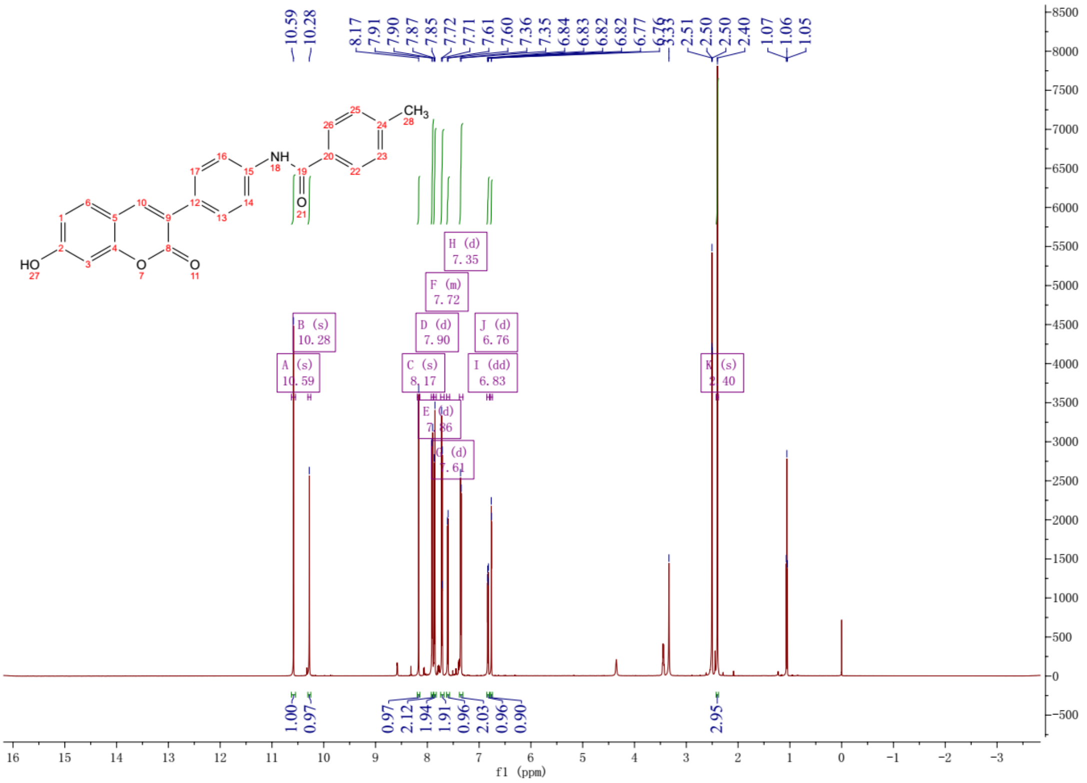

compound **4c** ^{13}CNMR

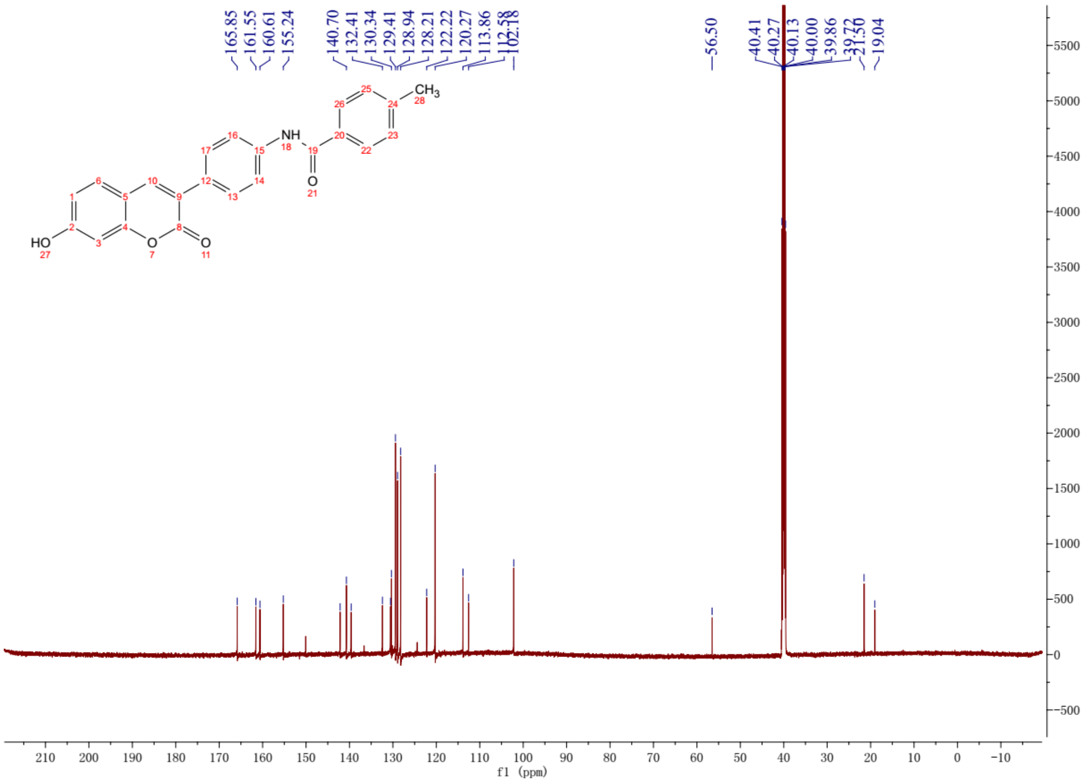

compound **4d** ^{1}HNMR

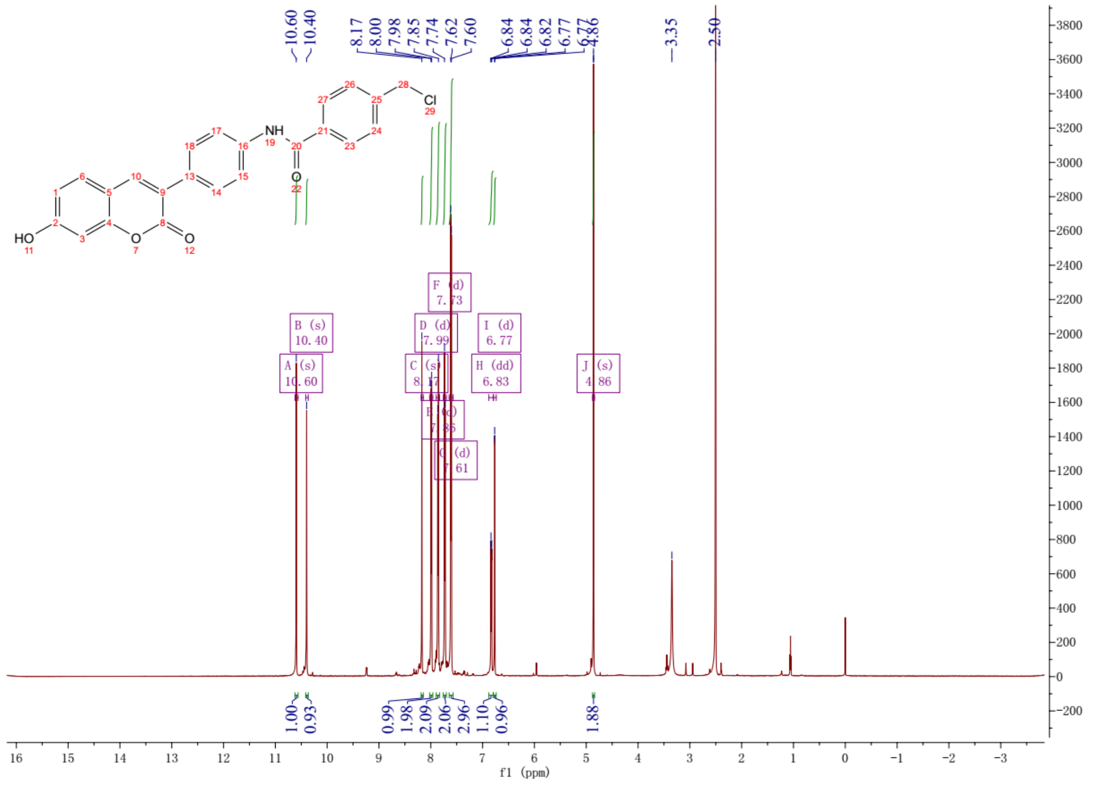

compound **4d** ^{13}CNMR

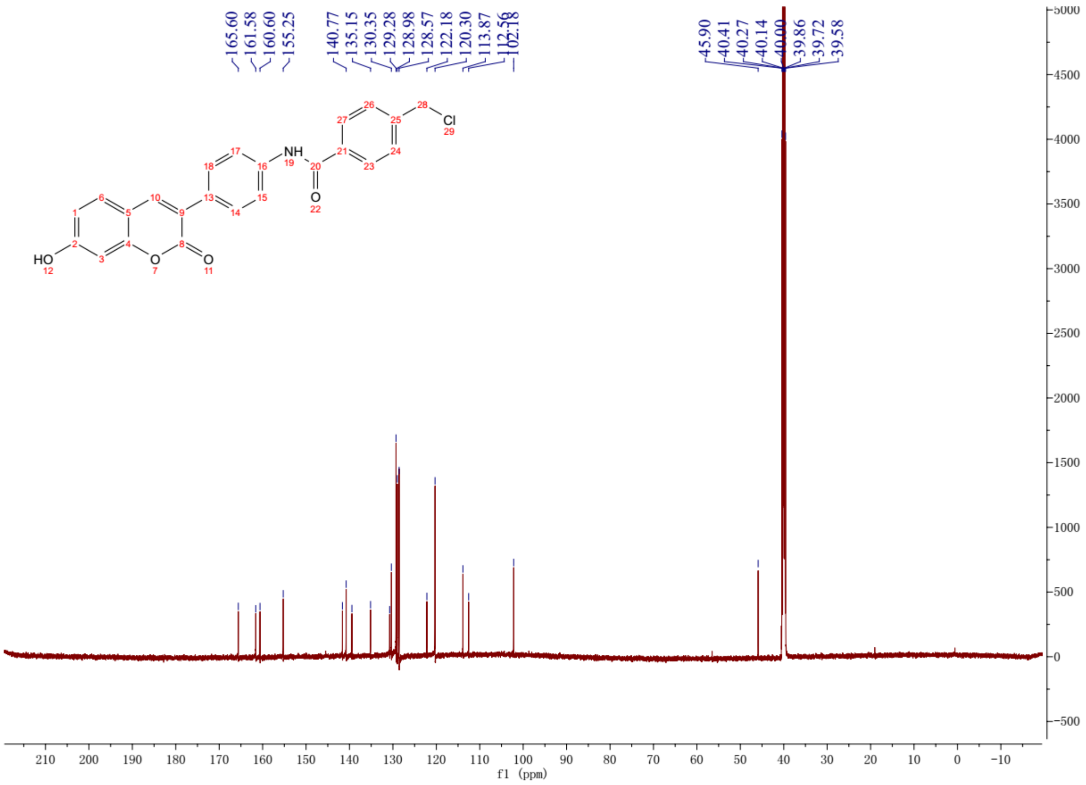

compound **4e** ^{1}HNMR

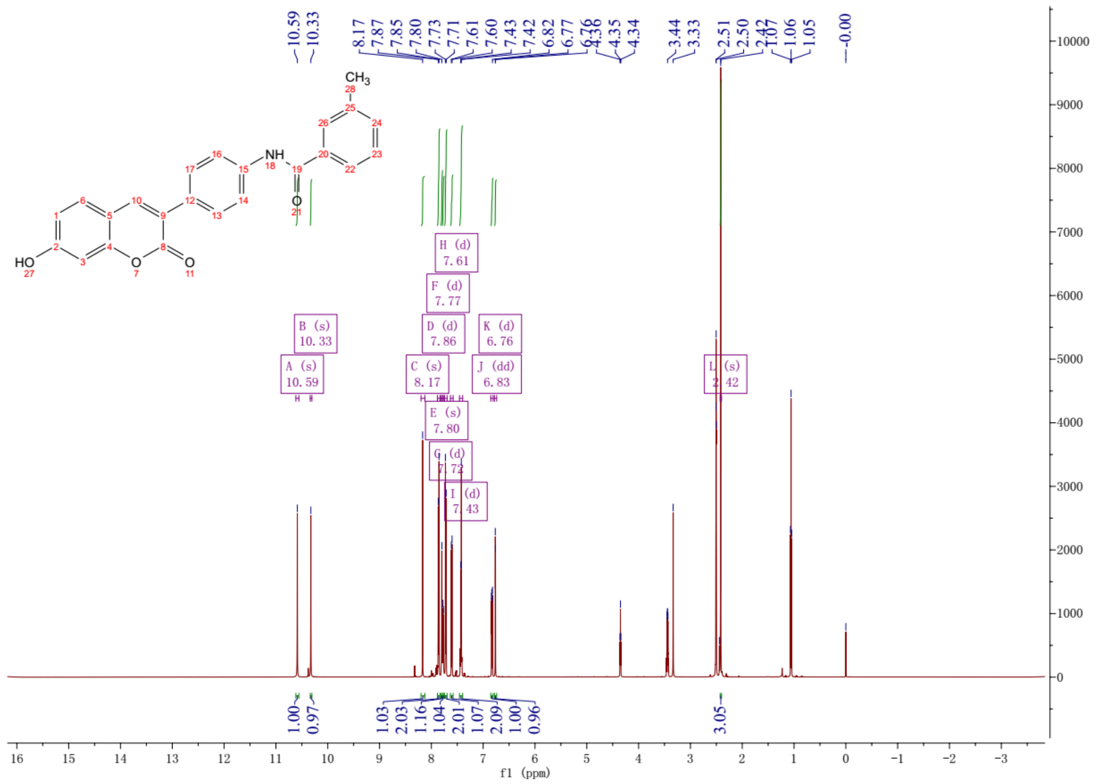

compound **4e** ^{13}CNMR

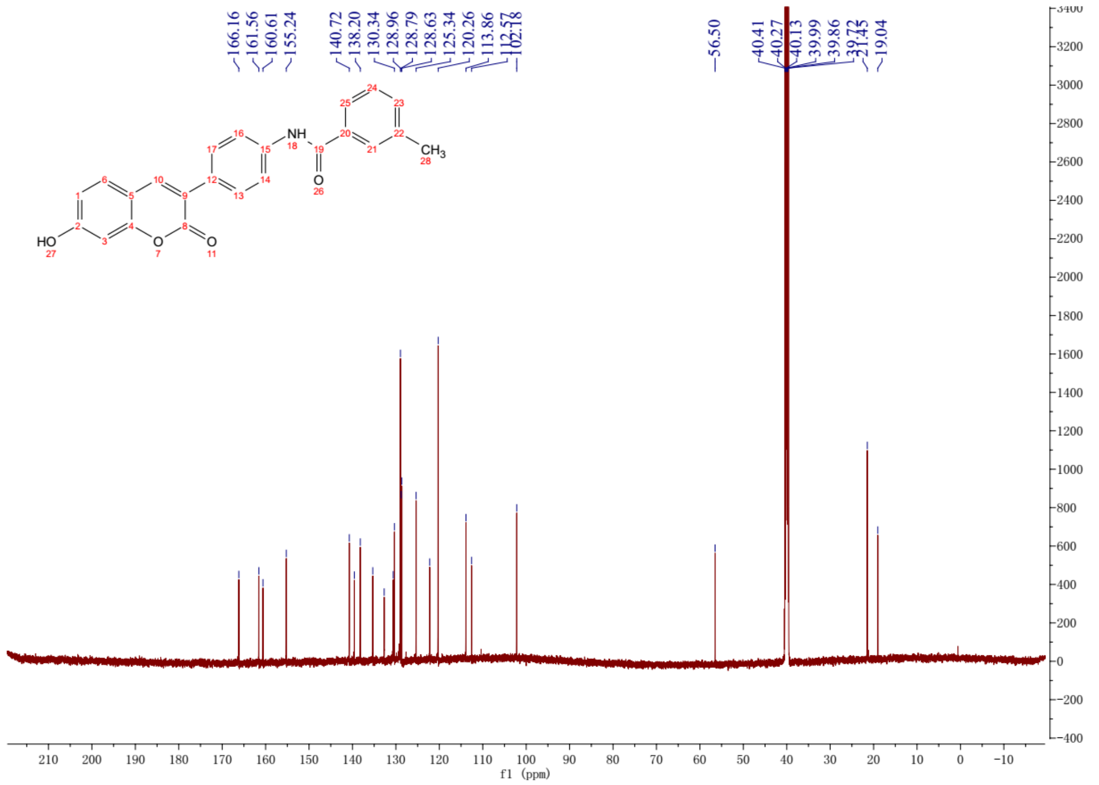

compound **4f** ^{1}HNMR

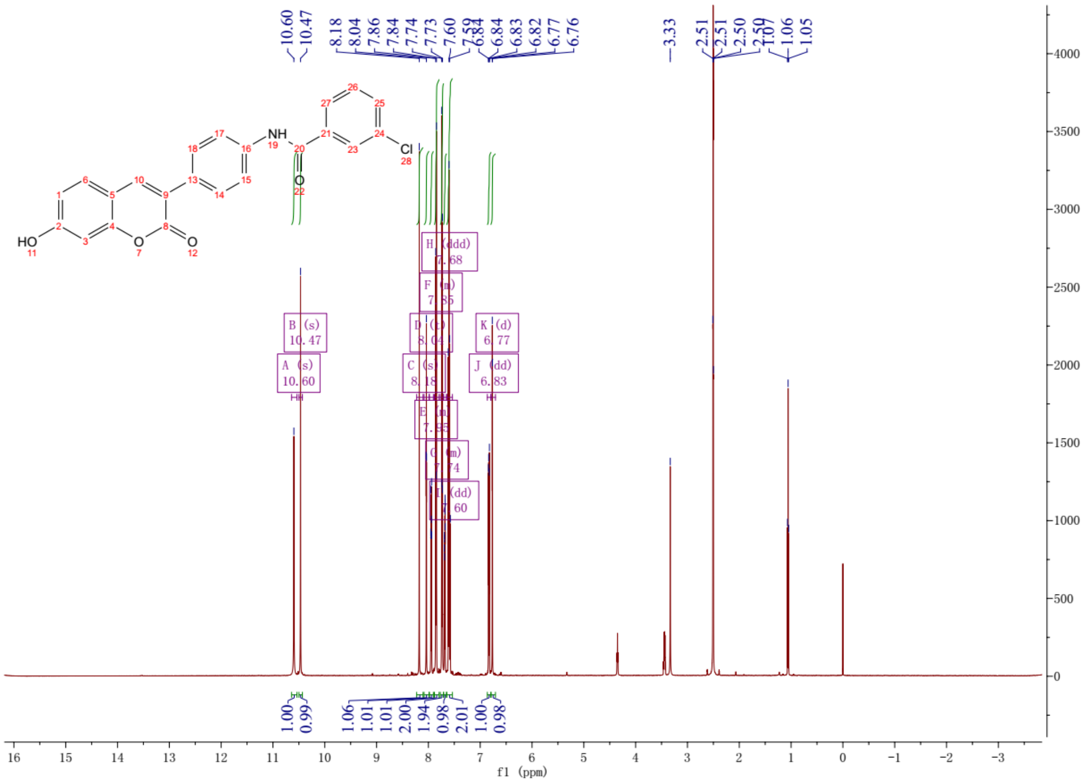

compound **4f** ^{13}CNMR

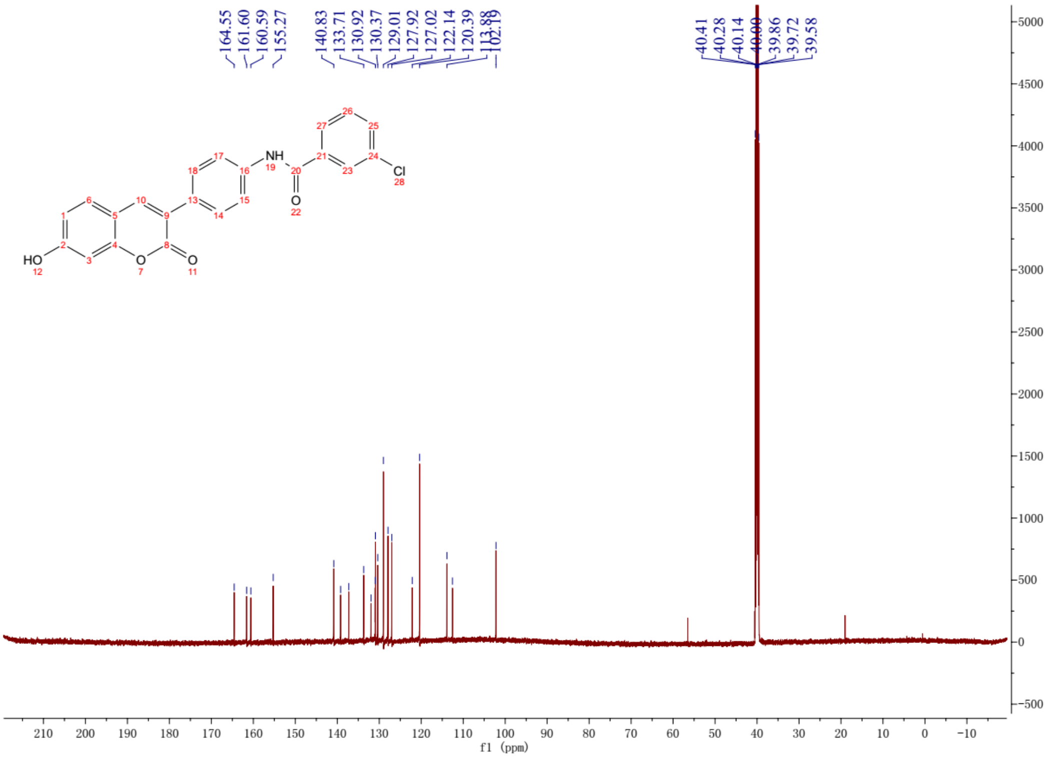

compound **4g** ^{1}HNMR

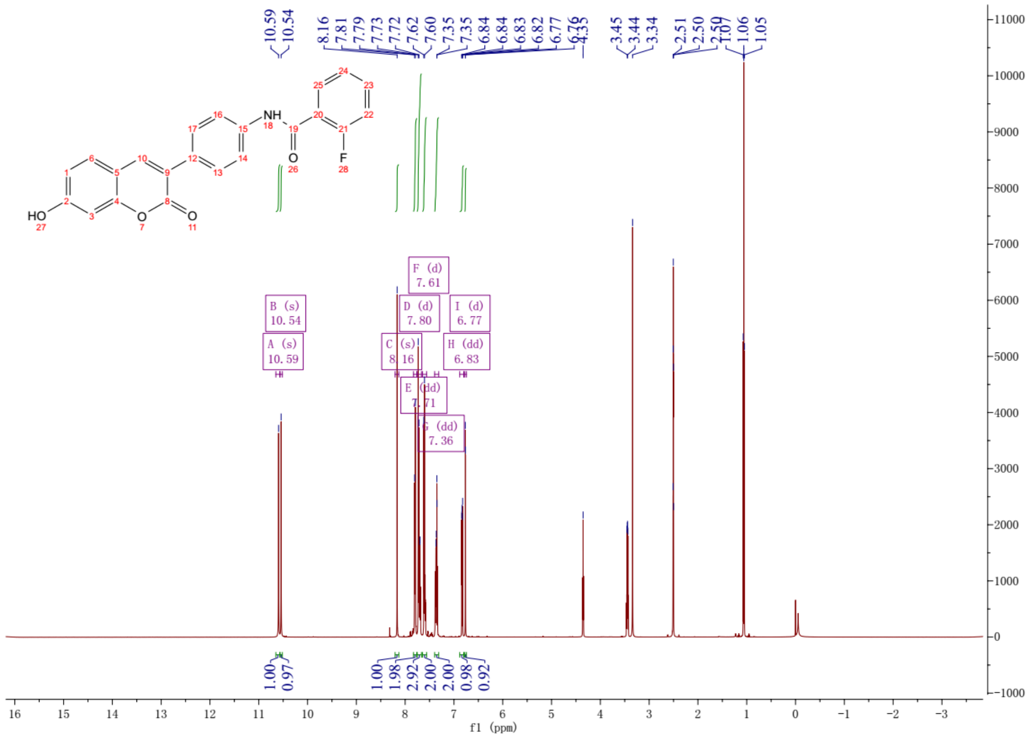

compound **4g** ^{13}CNMR

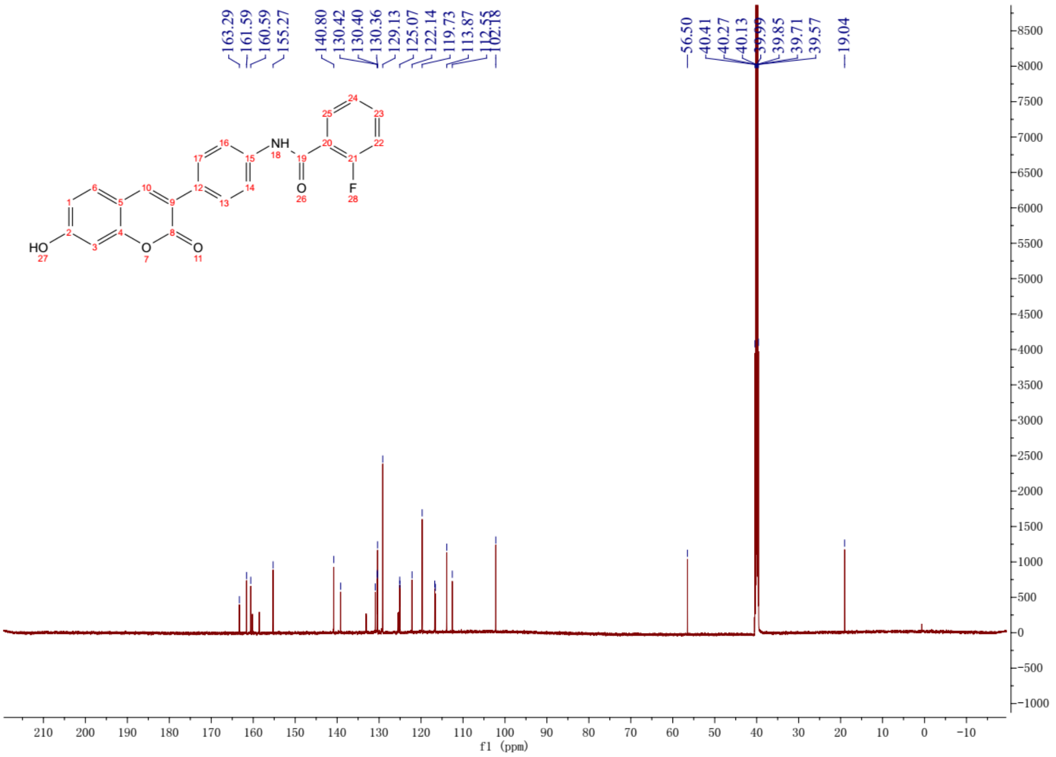

compound **4h** ^{1}HNMR

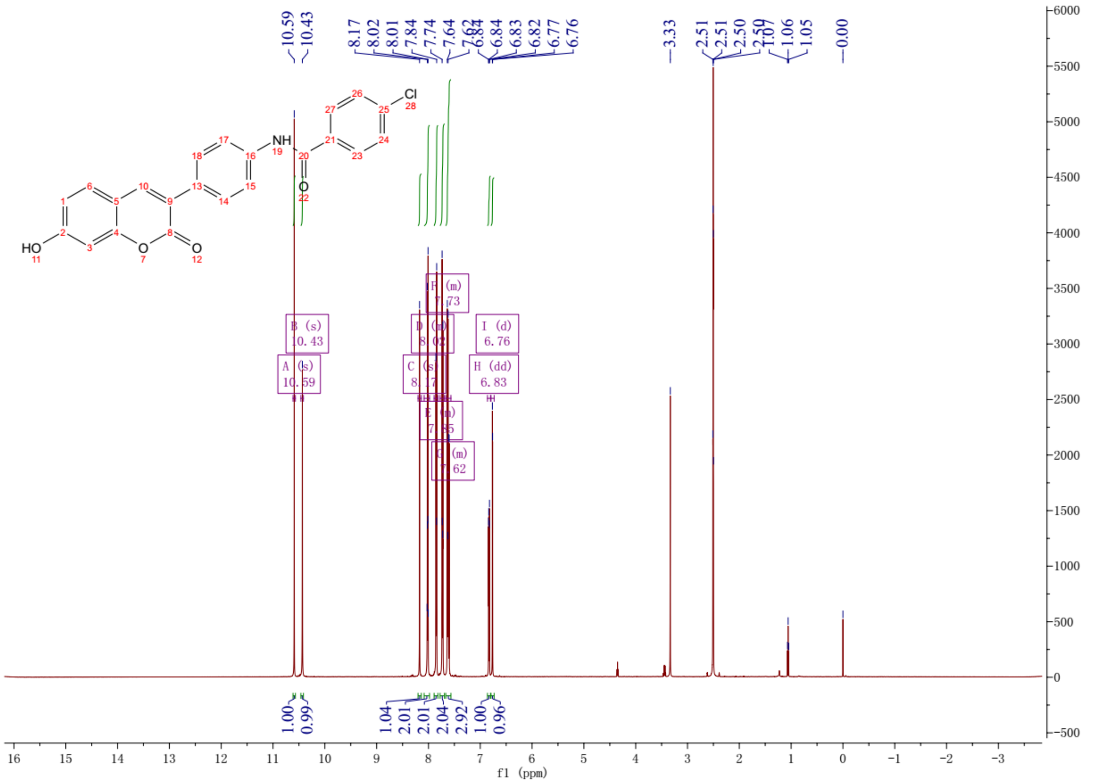

compound **4h** ^{13}CNMR

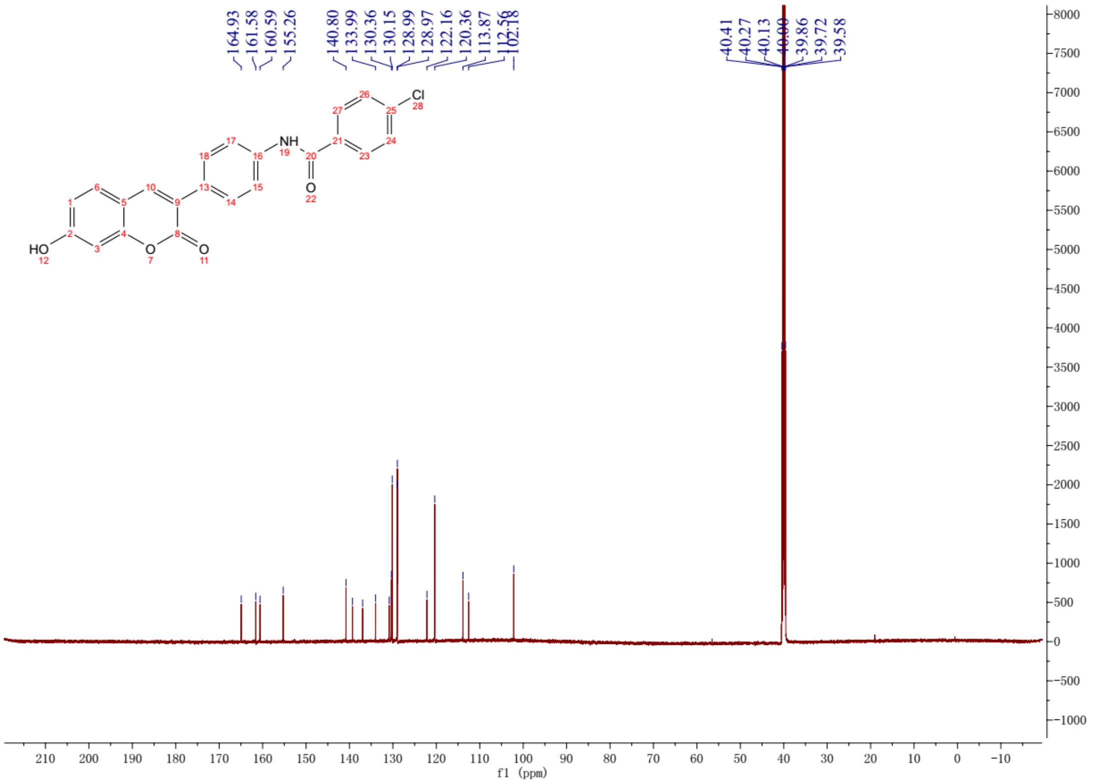

compound **4i** ^{1}HNMR

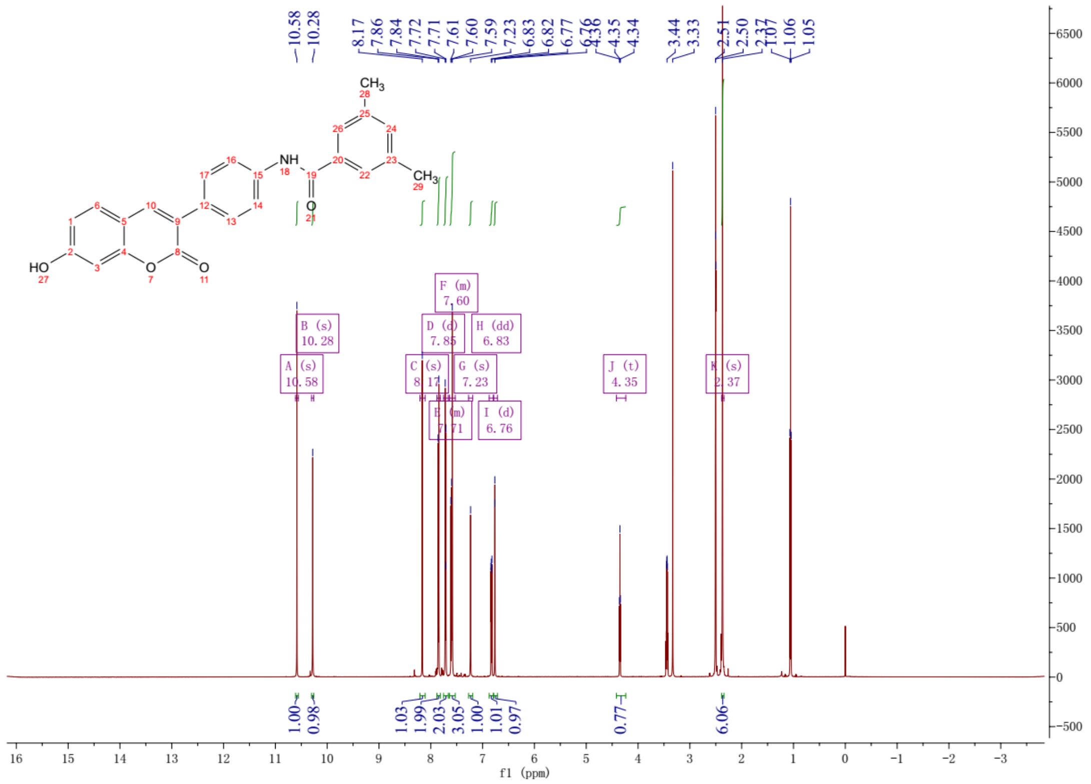

compound **4i** ^{13}CNMR

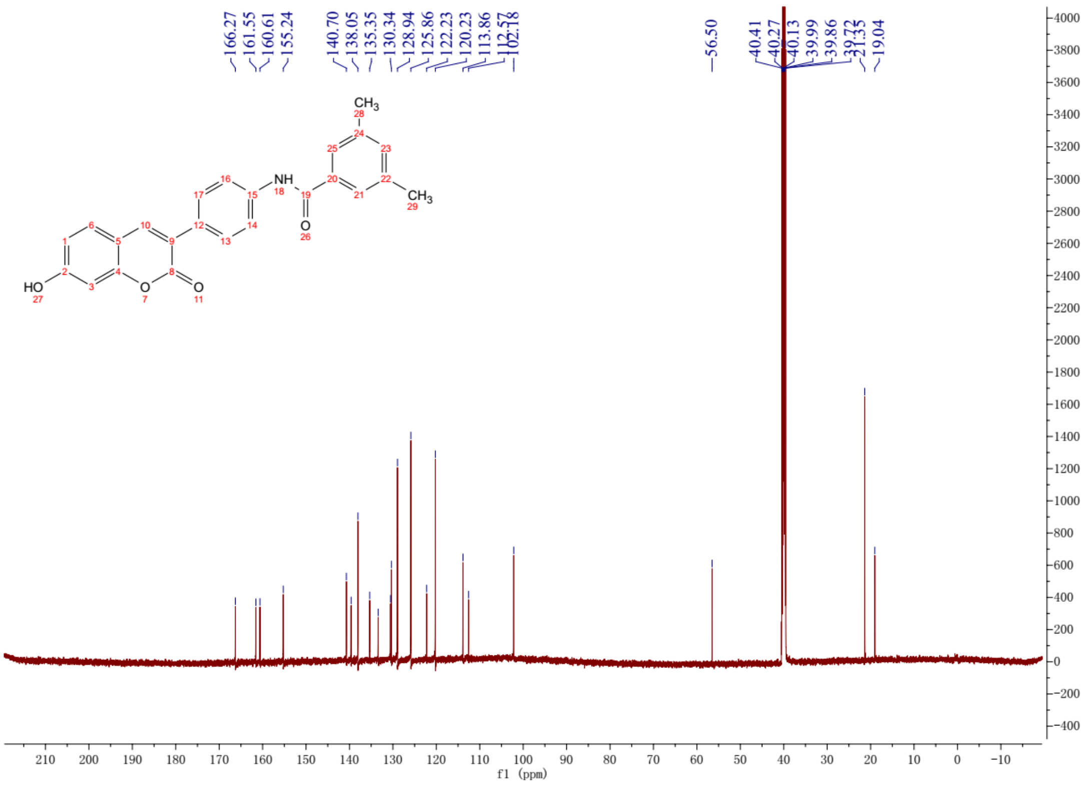

compound **4j** ^{1}HNMR

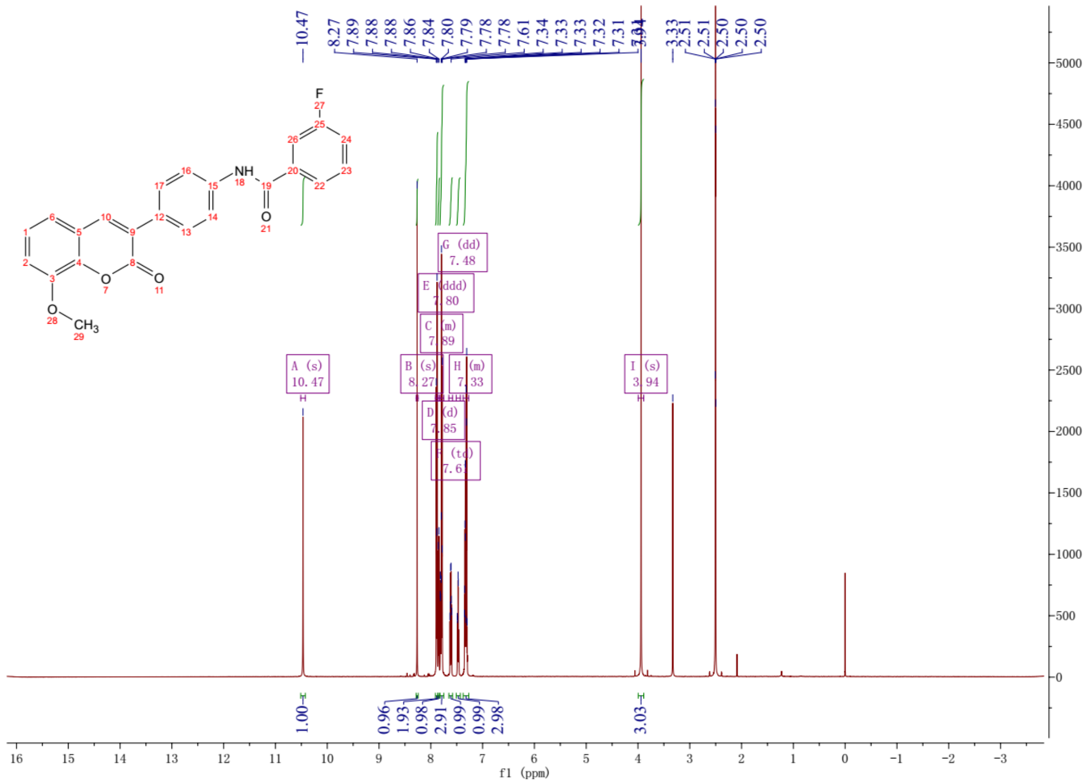

compound **4j** ^{13}CNMR

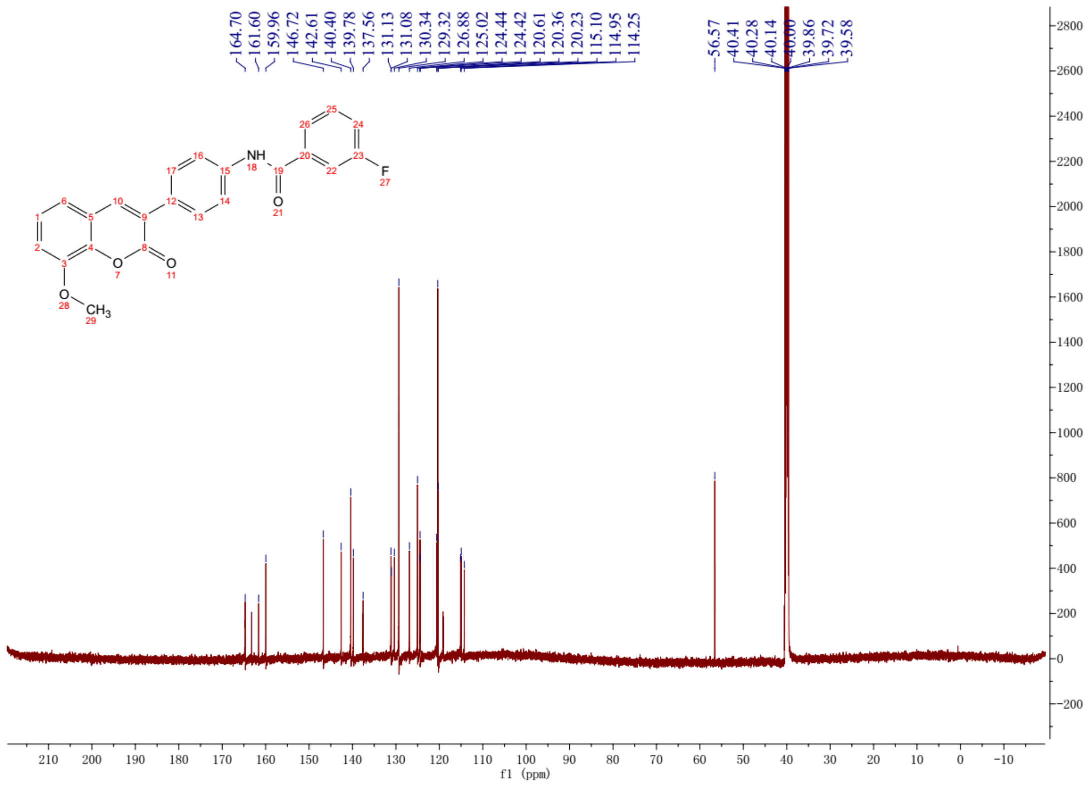

compound **4k** ^{1}HNMR

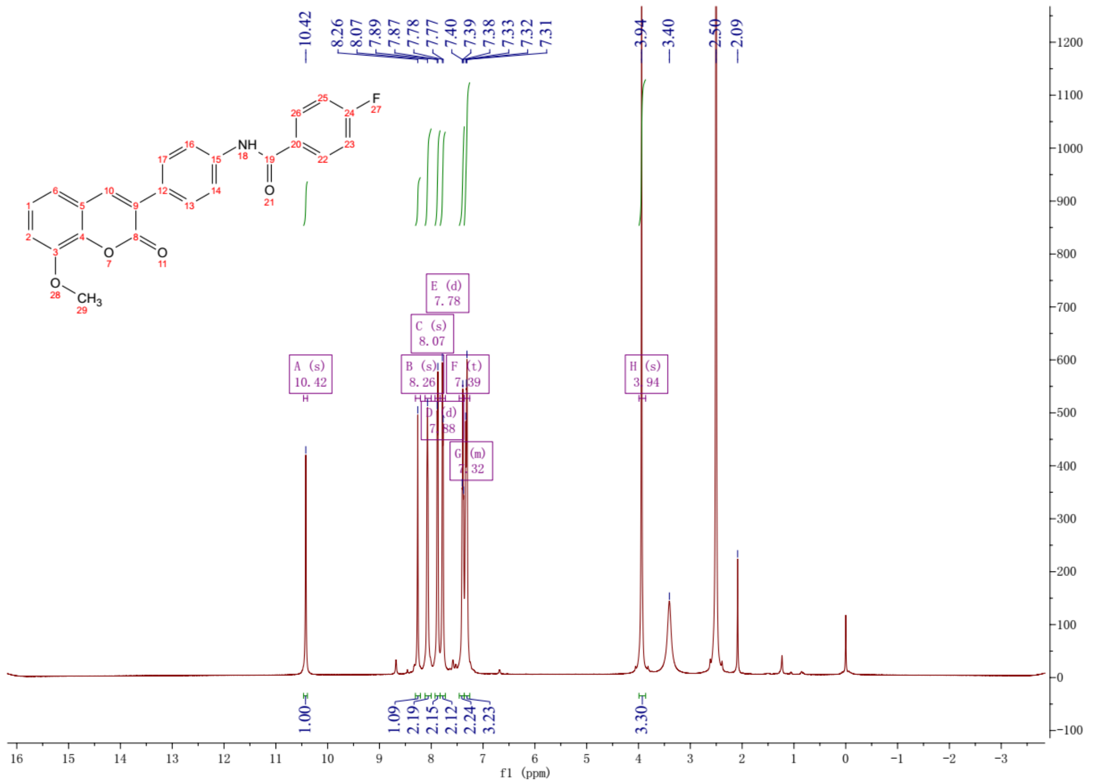

compound **4k** ^{13}CNMR

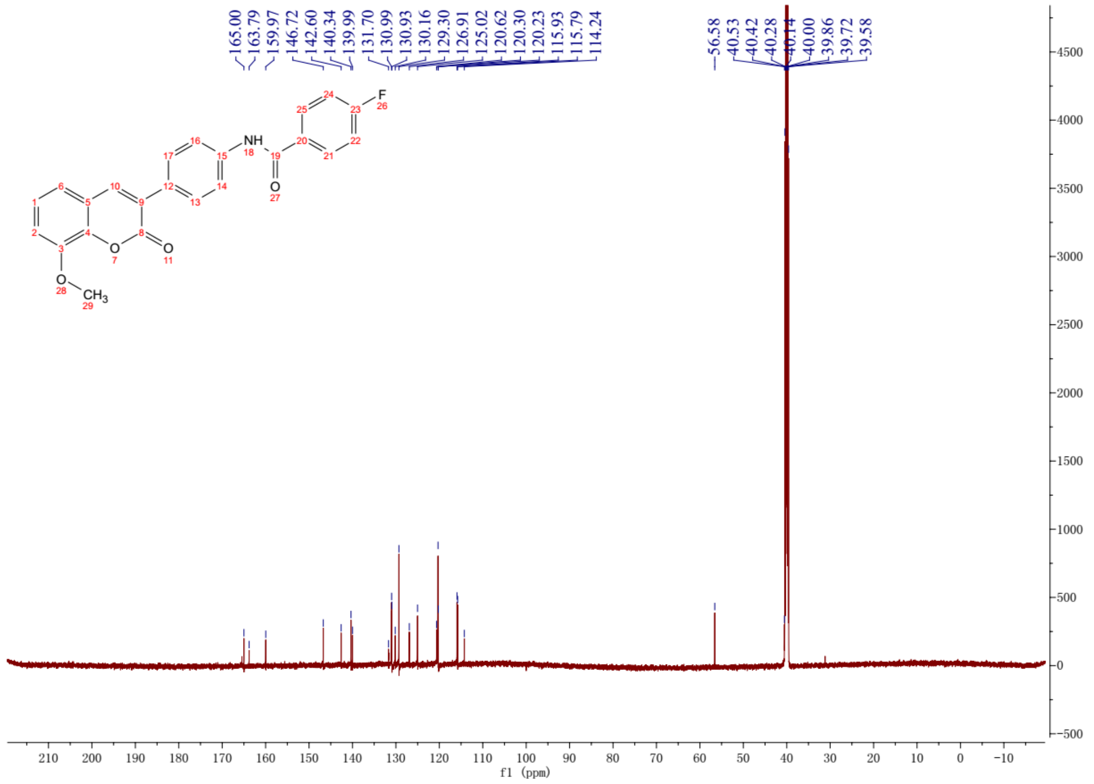

compound **4l** ^{1}HNMR

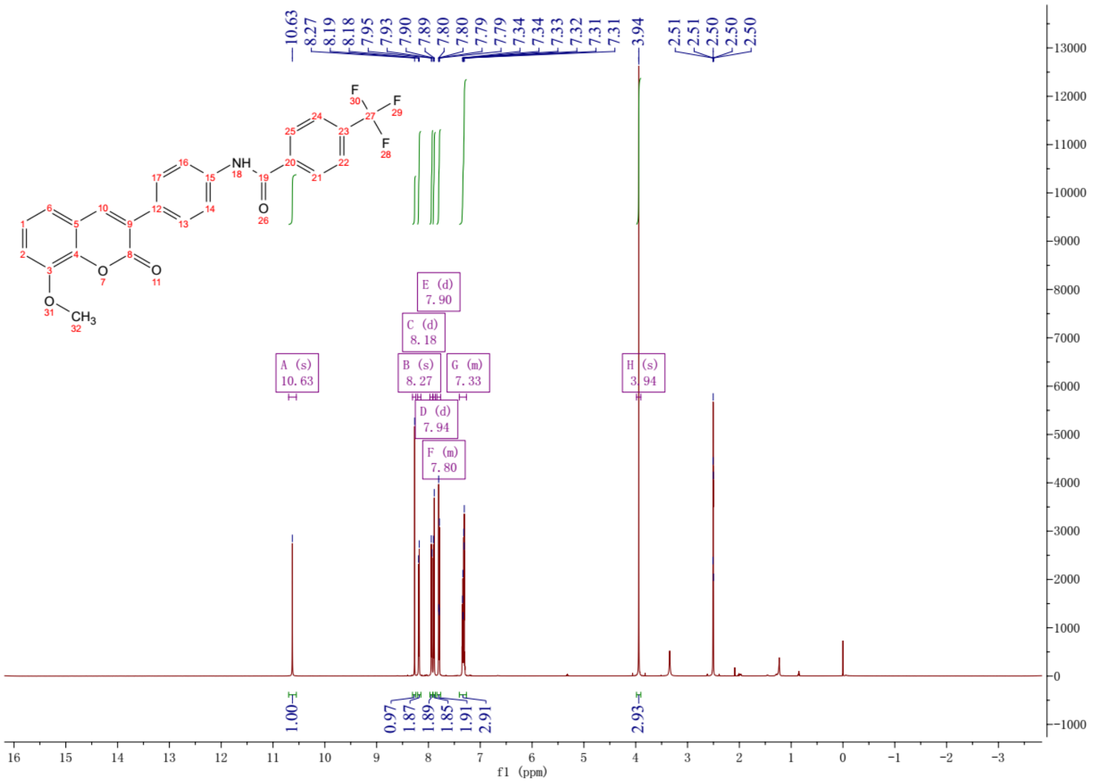

compound **4l** ^{13}CNMR

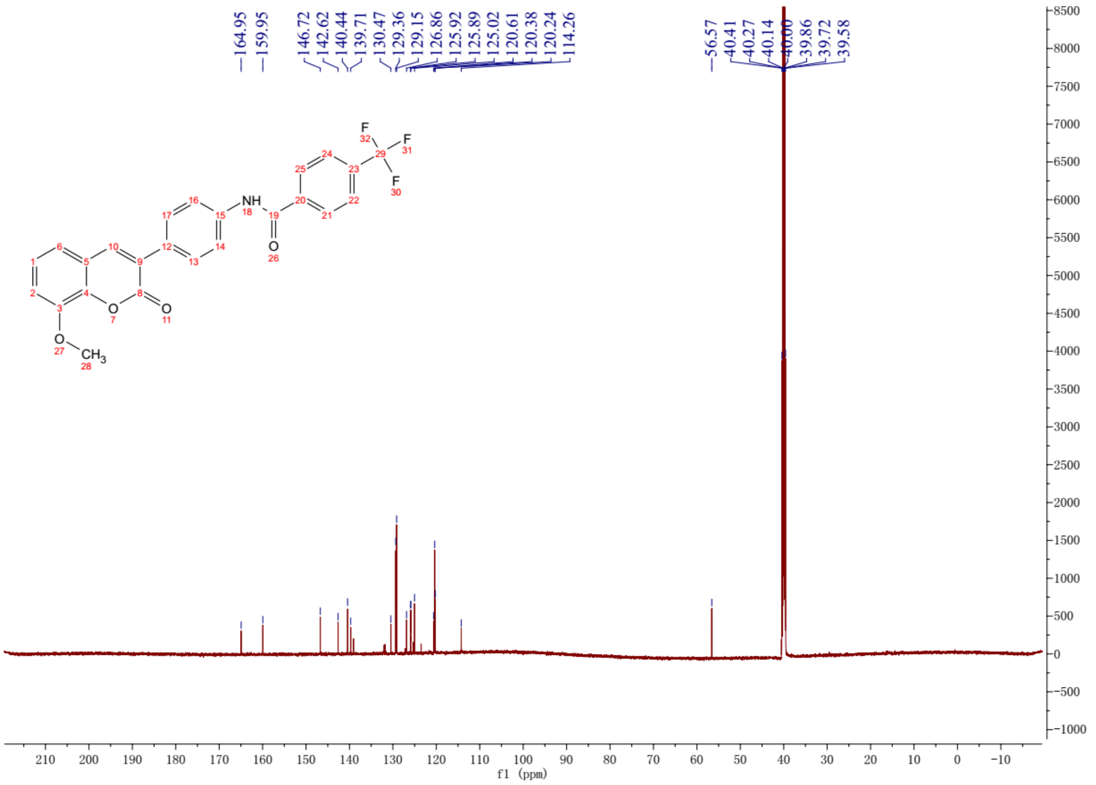

compound **4m** ^{1}HNMR

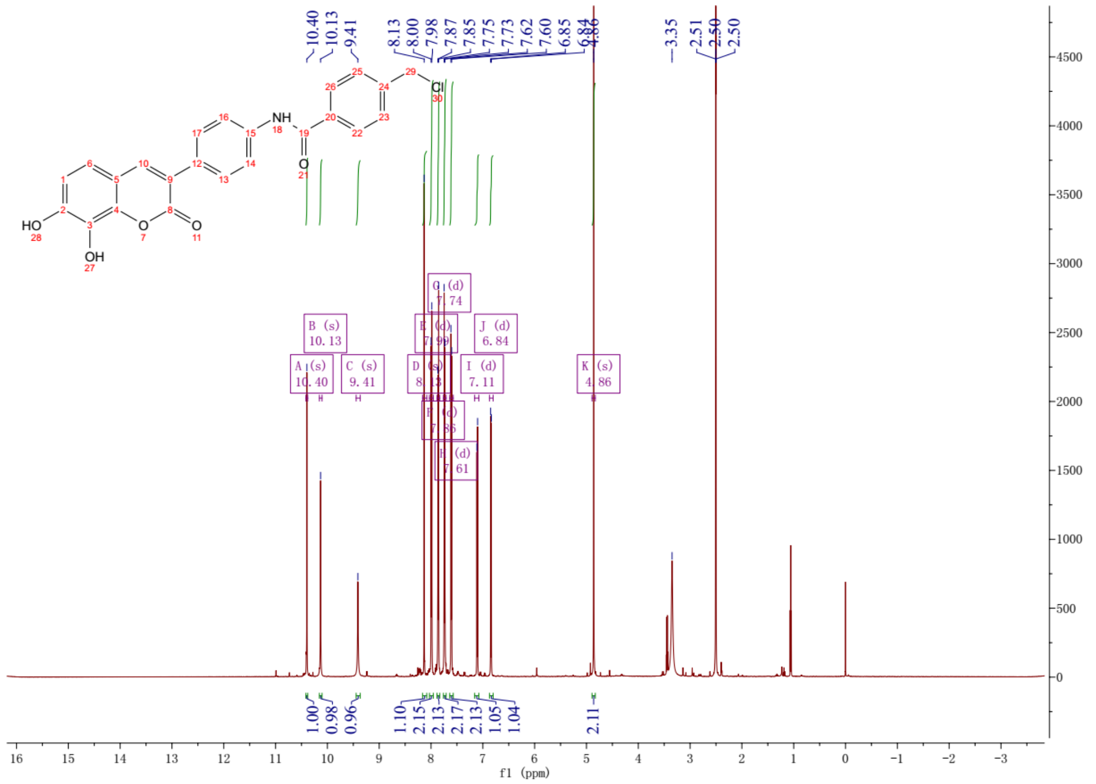

compound **4m** ^{13}CNMR

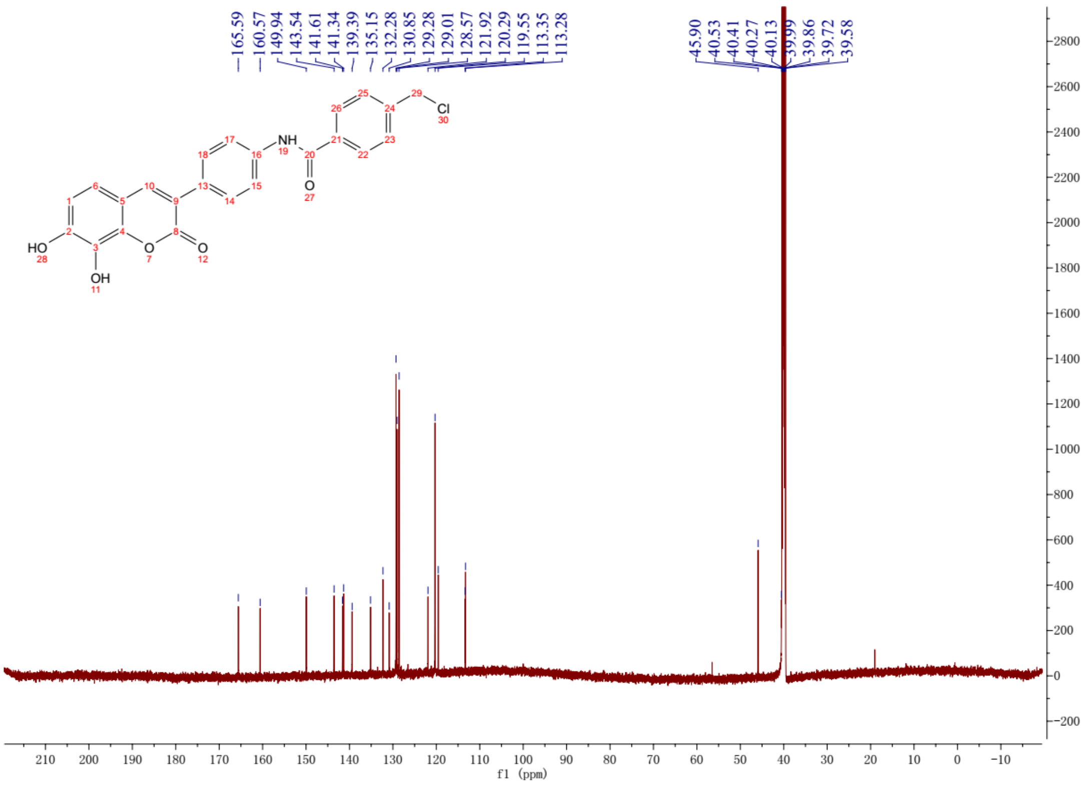

compound **4n** ^{1}HNMR

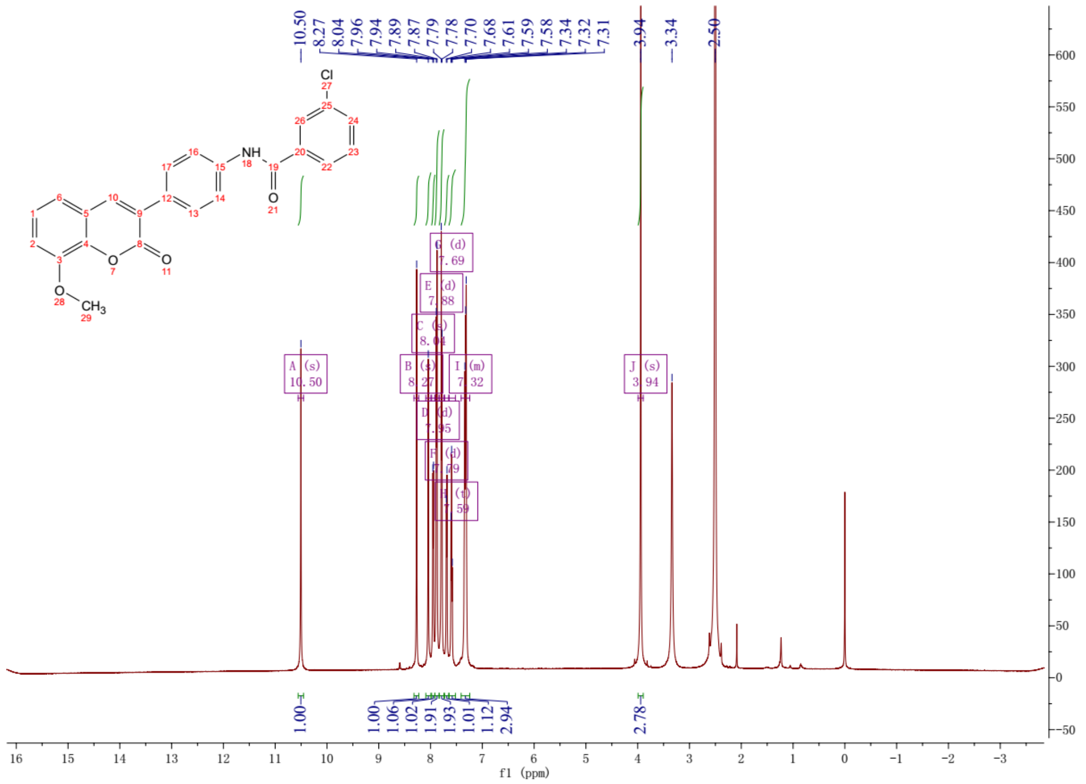

compound **4n** ^{13}CNMR

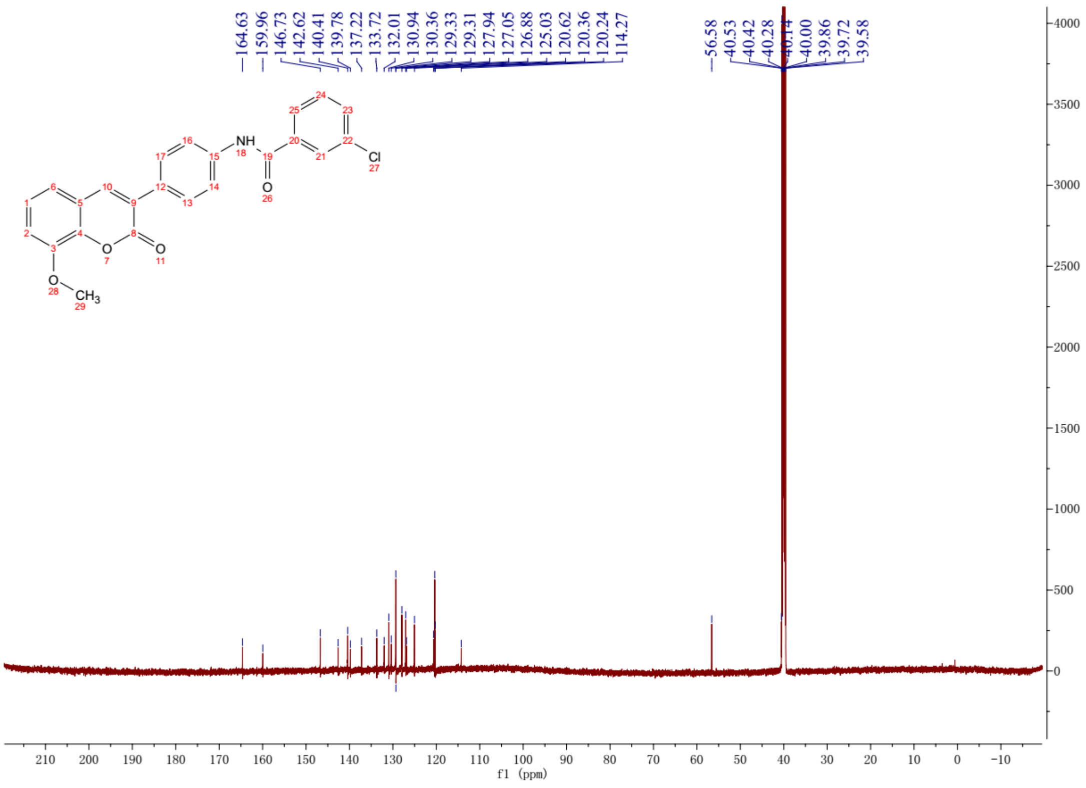

compound **4o** ^{1}HNMR

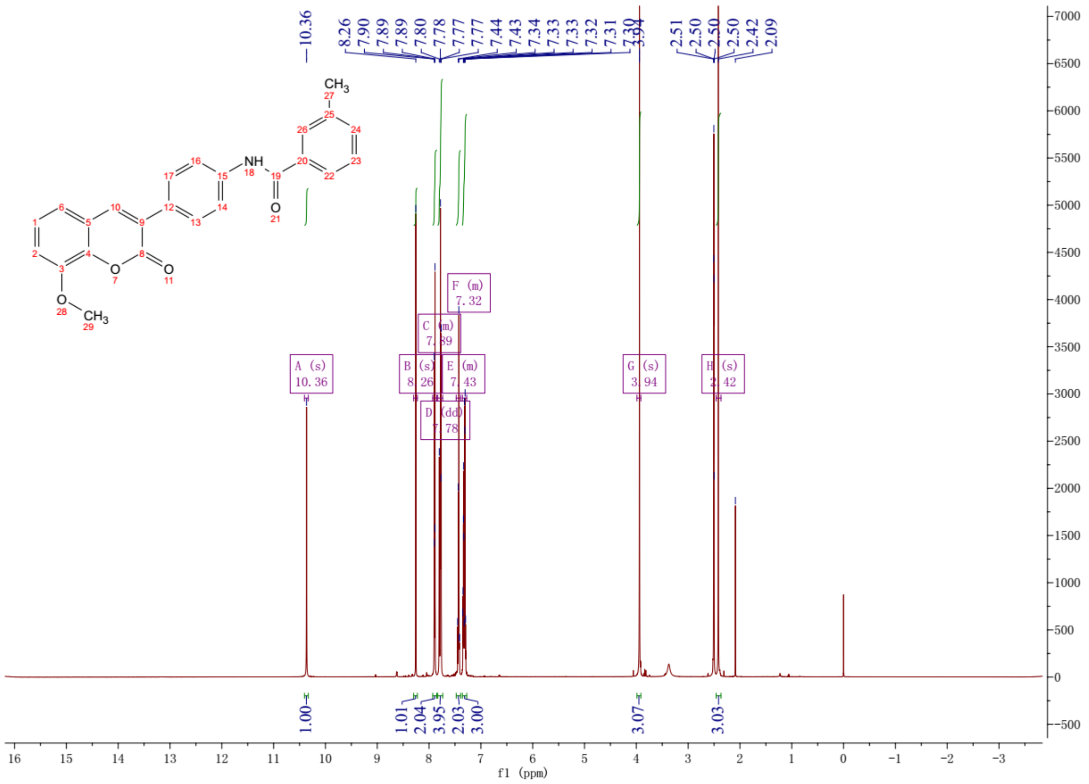

compound **4o** ^{13}CNMR

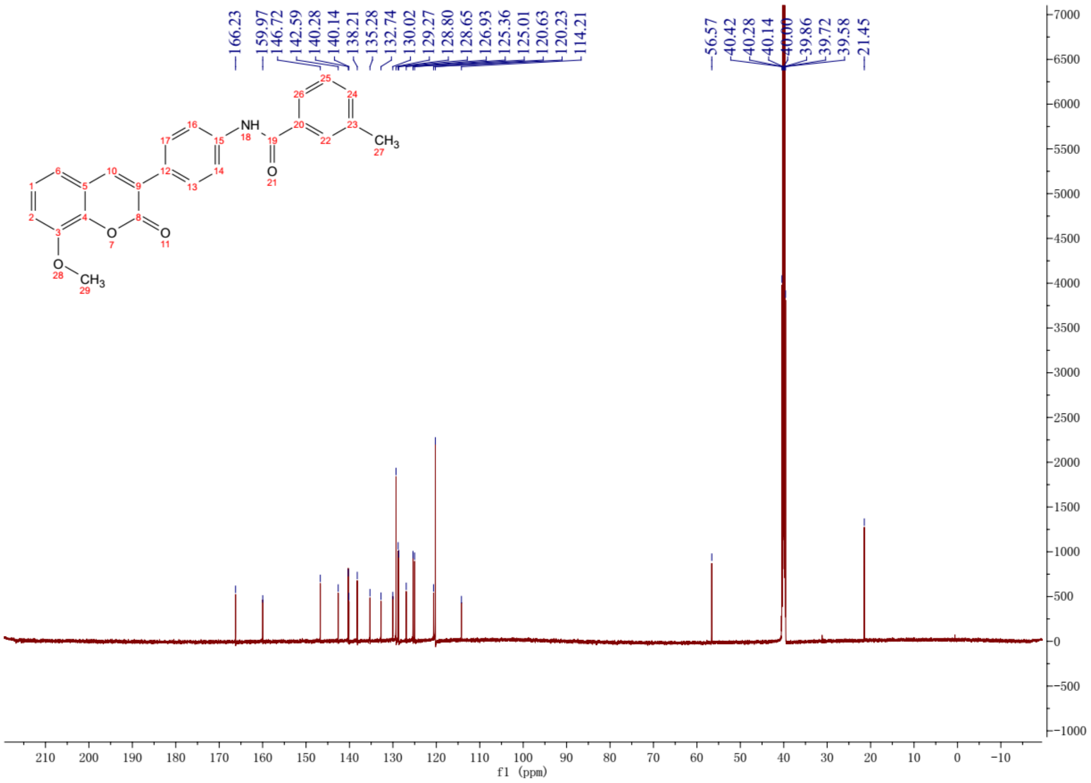

compound **4p** ^{1}HNMR

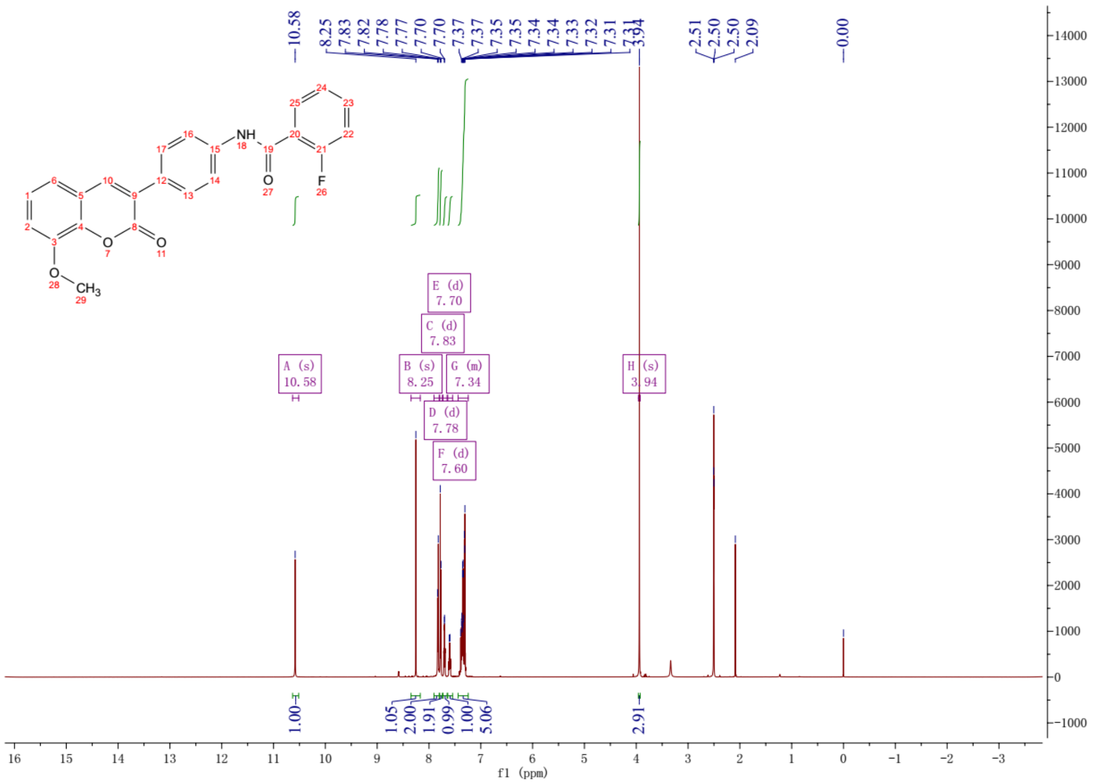

compound **4p** ^{13}CNMR

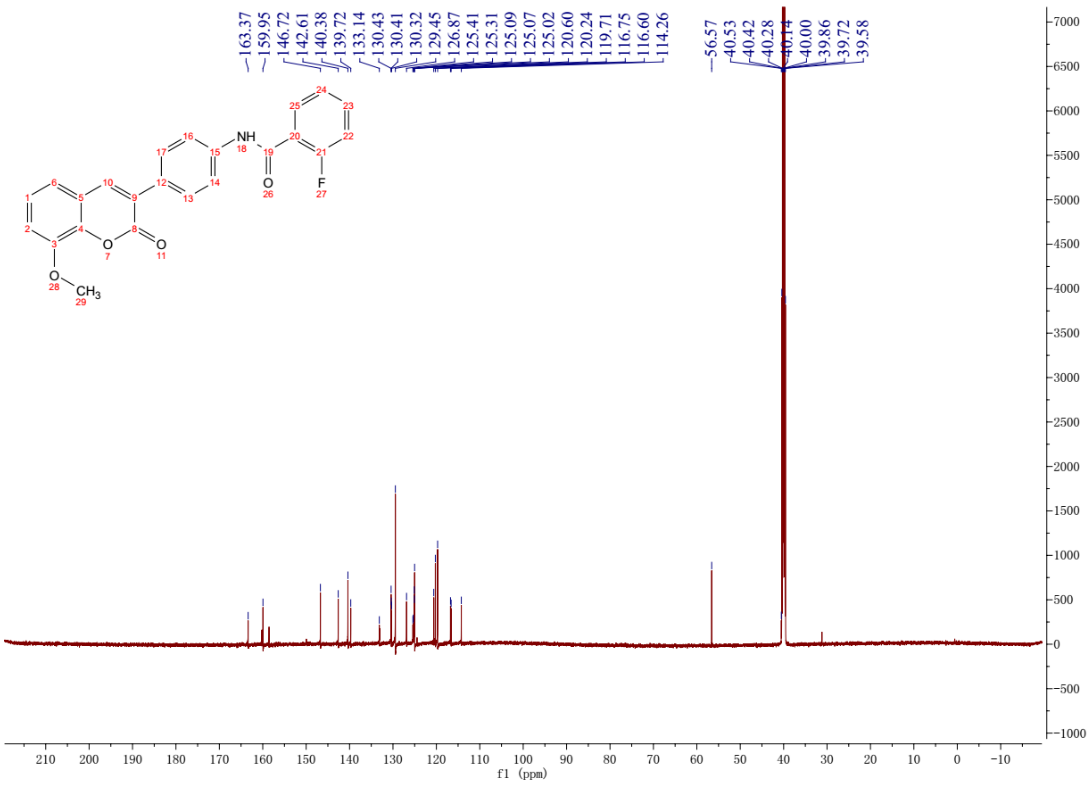

compound **4q** ^{1}HNMR

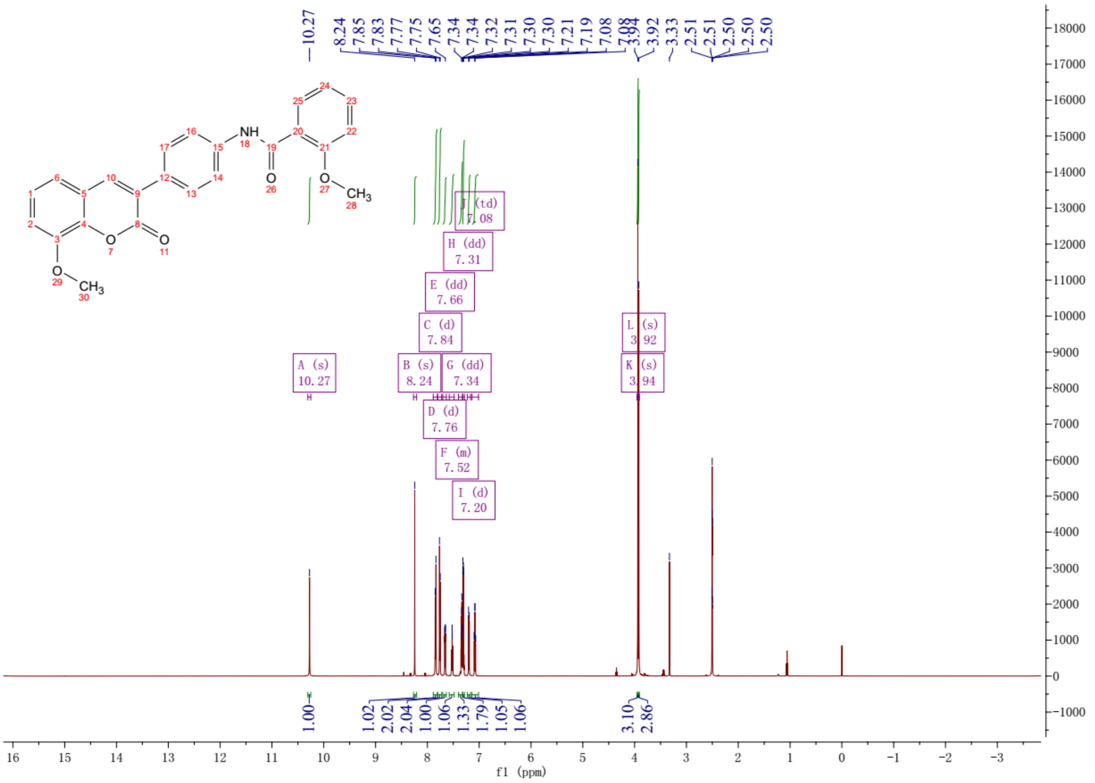

compound **4q** ^{13}CNMR

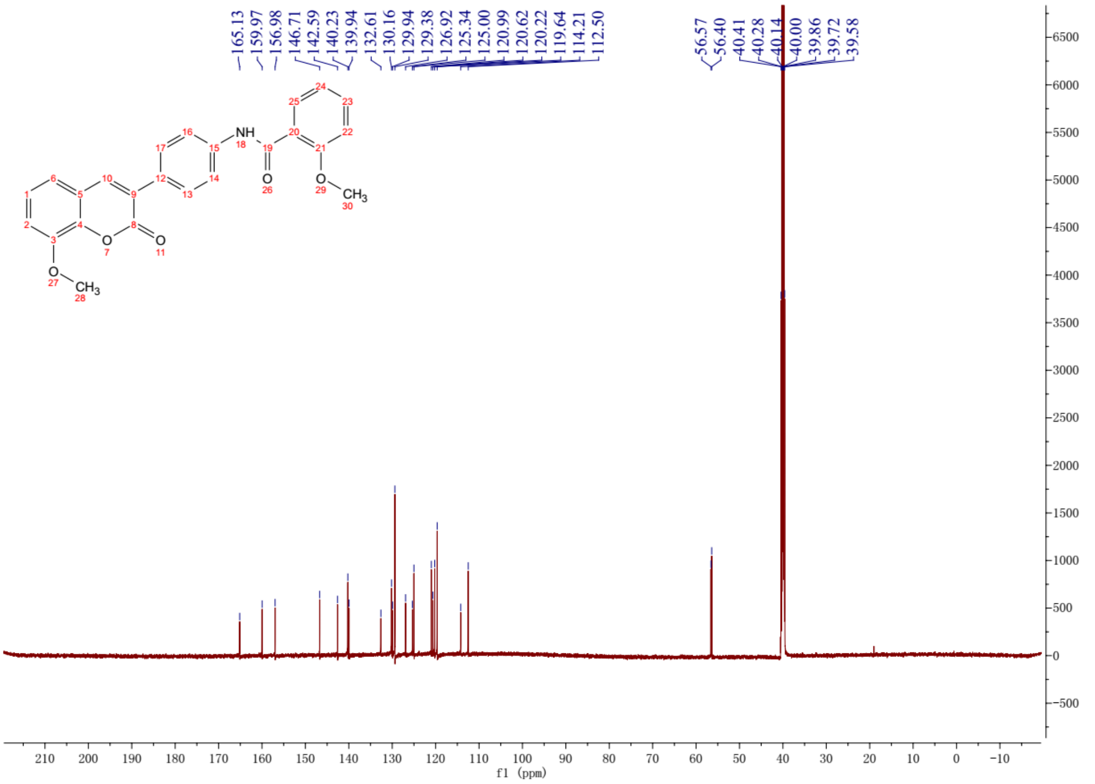

compound **4r** ^{1}HNMR

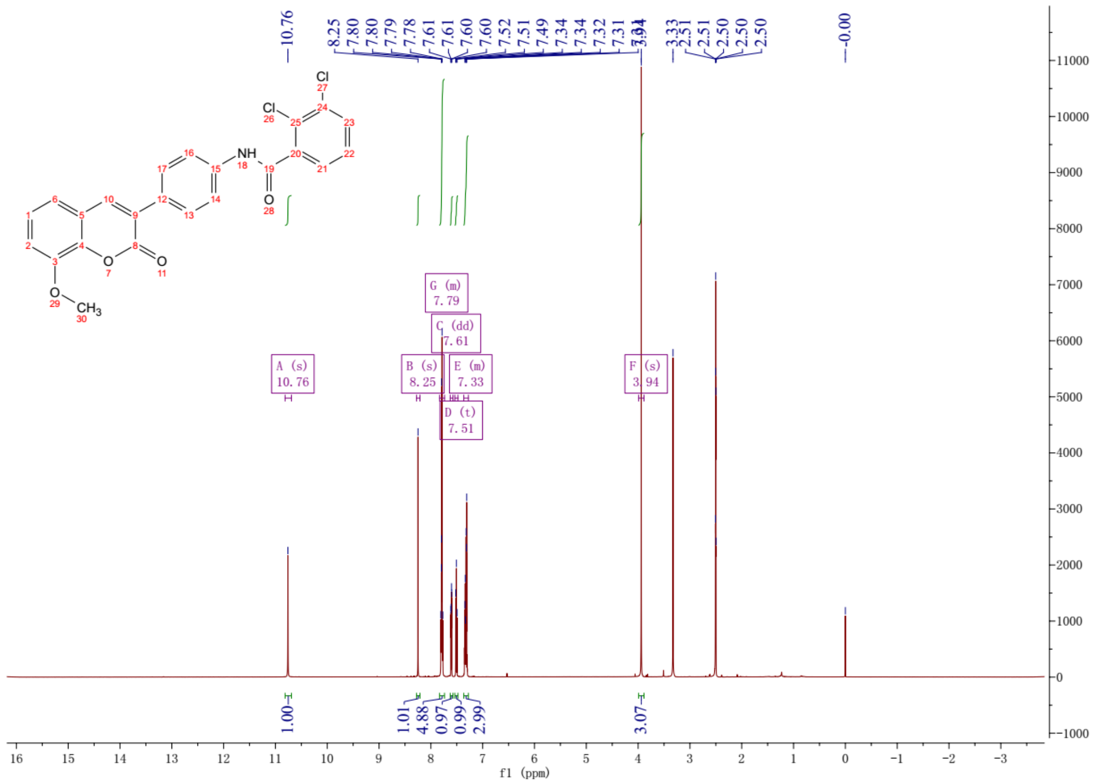

compound **4r** ^{13}CNMR

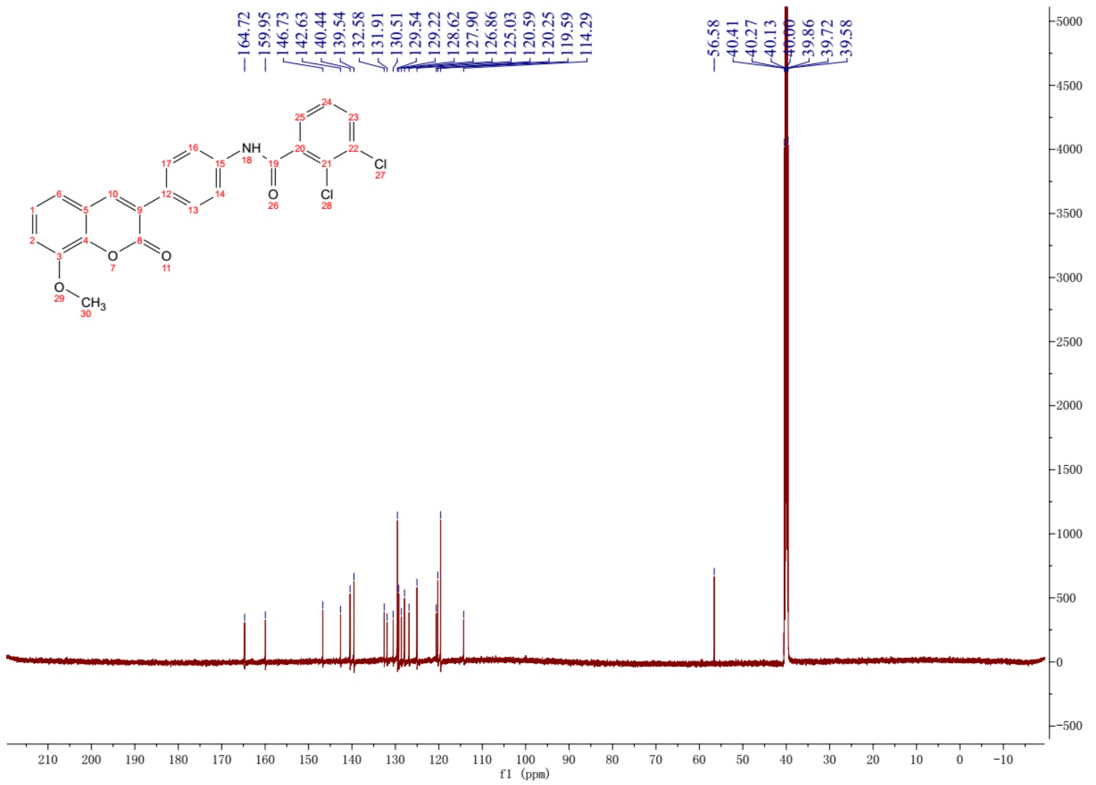

compound **4s** ^{1}HNMR

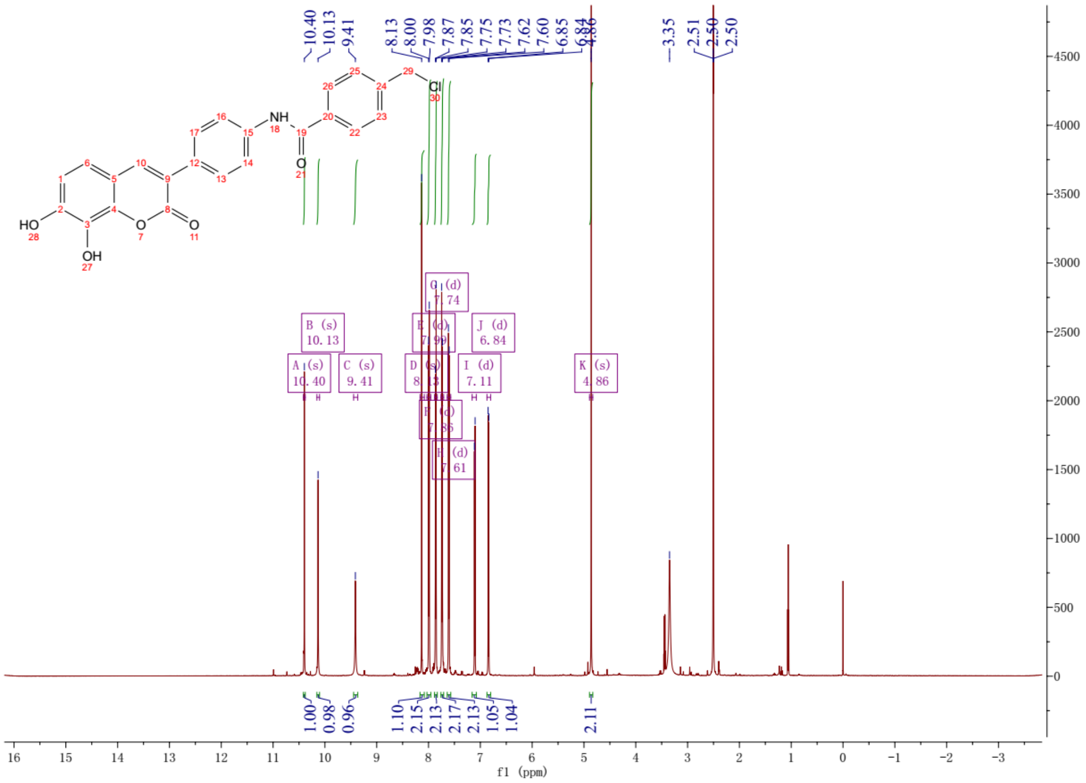

compound **4s** ^{13}CNMR

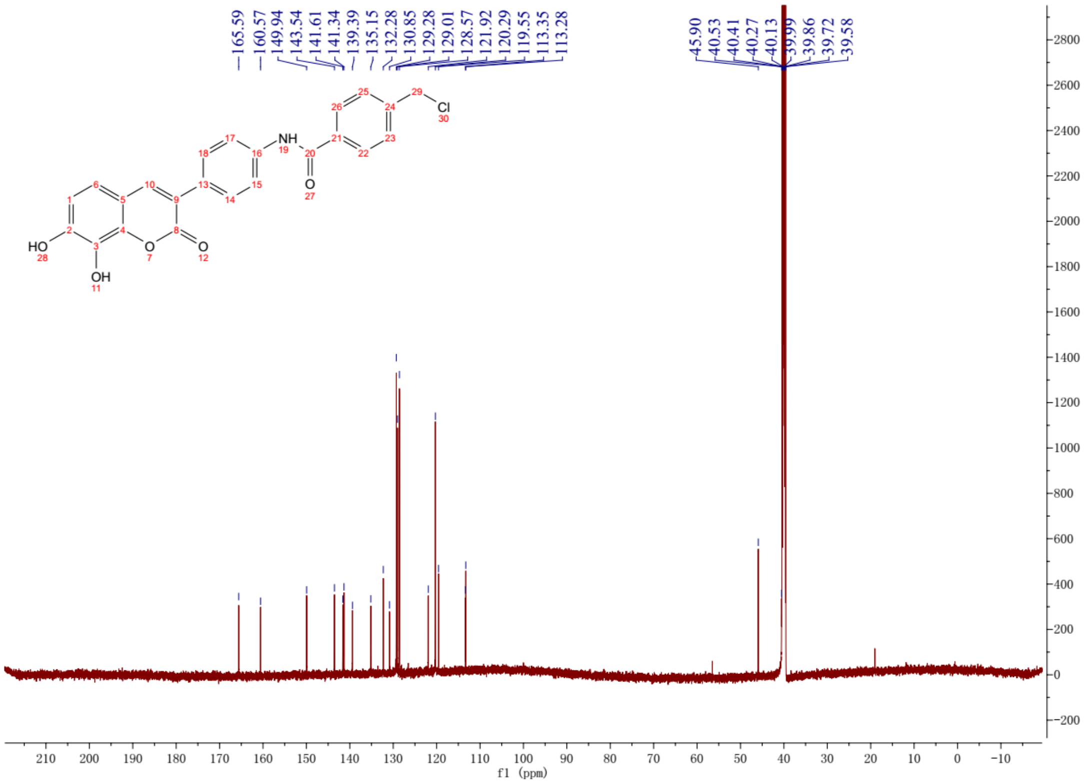

compound **5a** ^{1}HNMR

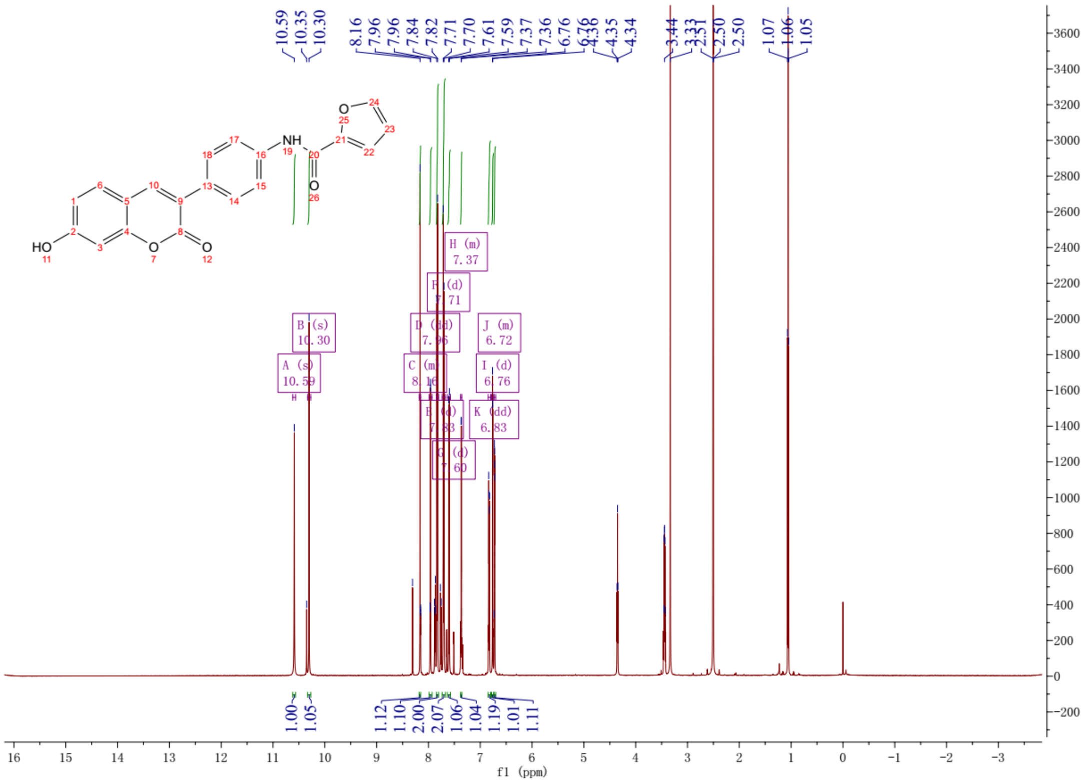

compound **5a** ^{13}CNMR

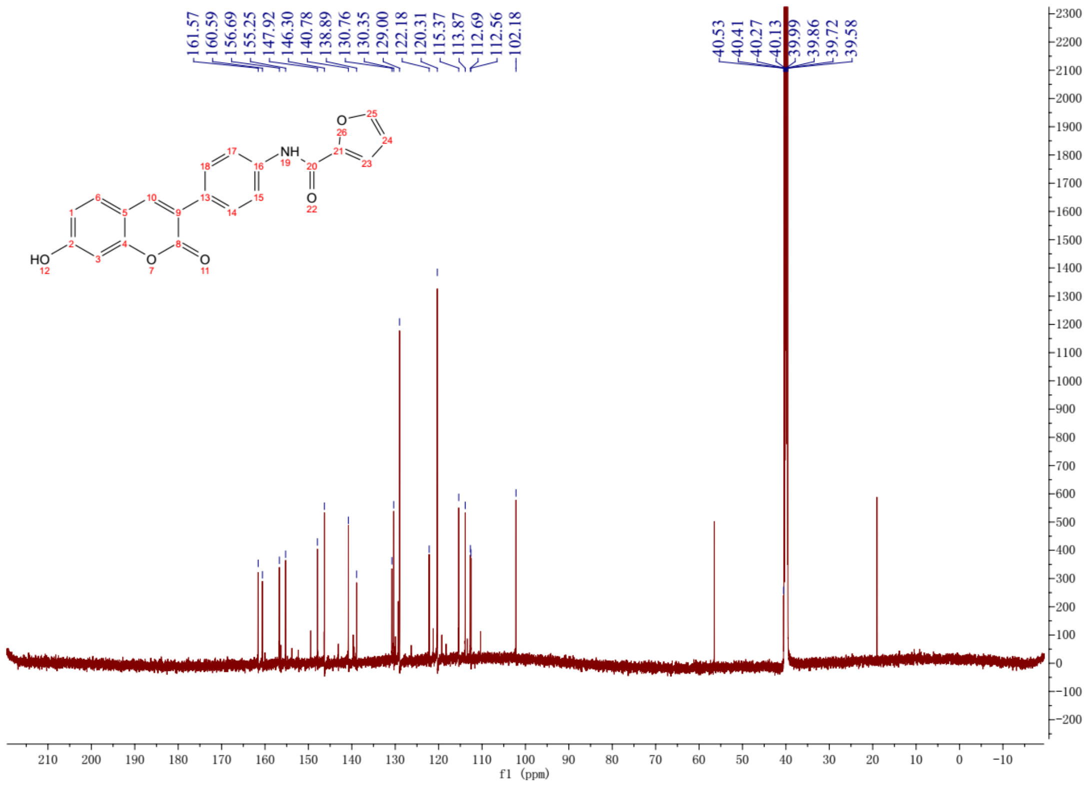

compound **5b** ^{1}HNMR

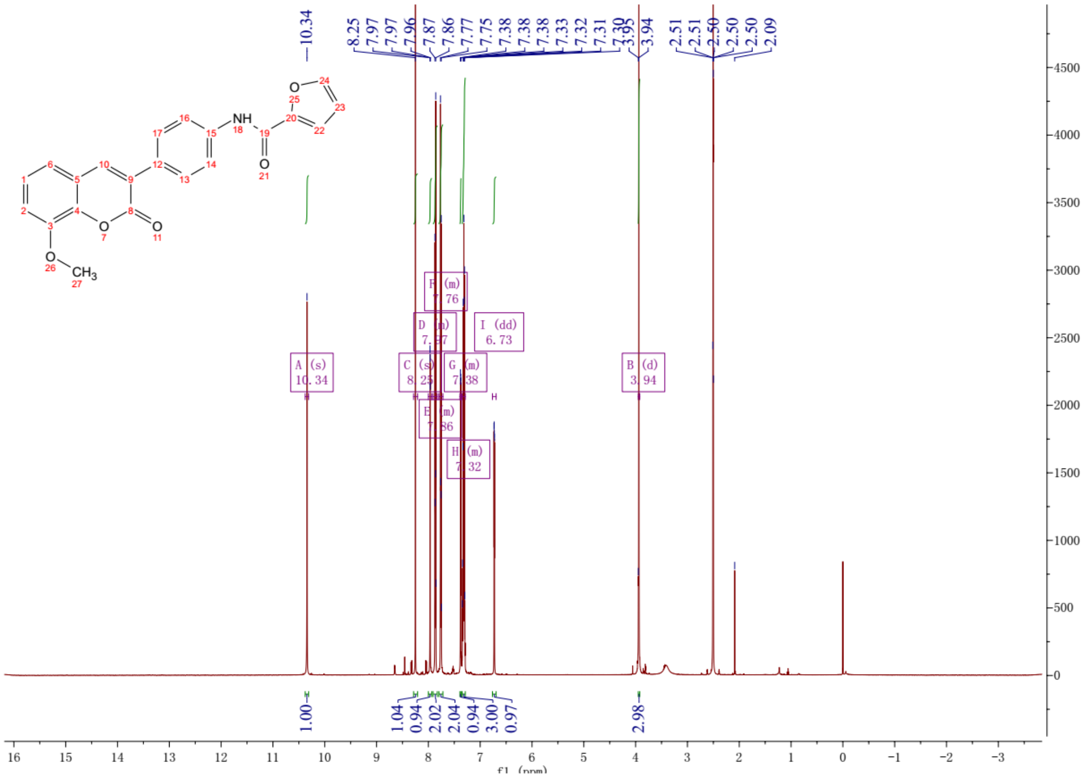

compound **5b** ^{13}CNMR

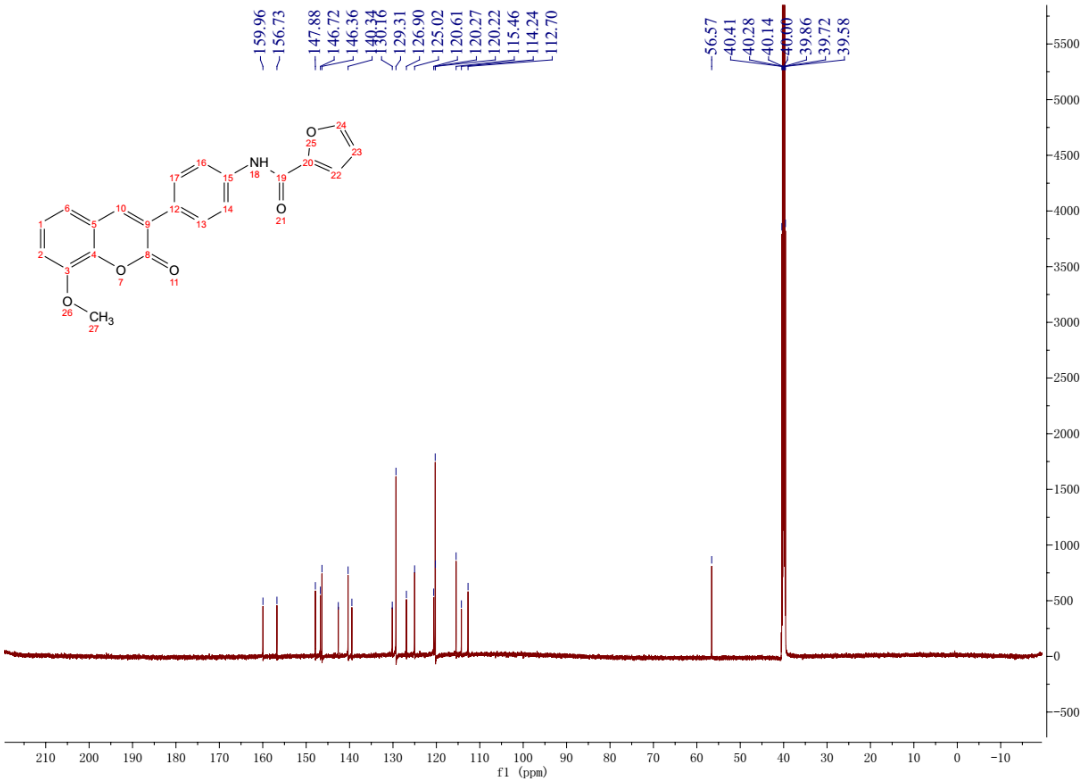

compound **6a** ^{1}HNMR

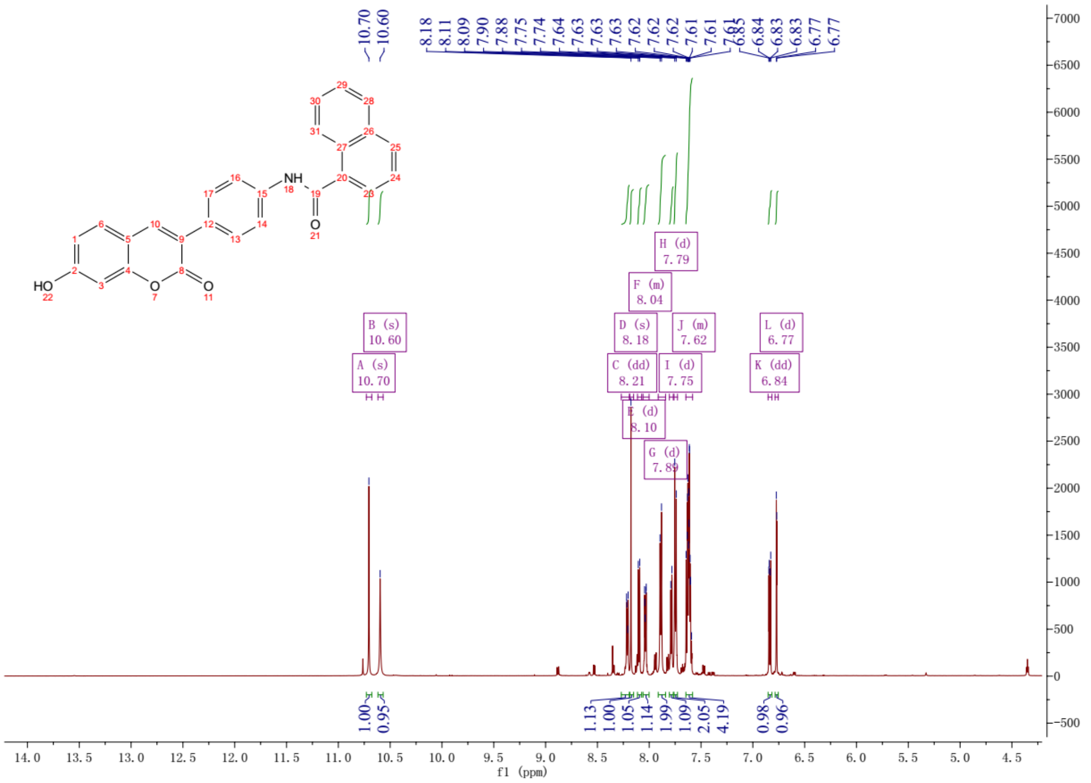

compound **6a** ^{13}CNMR

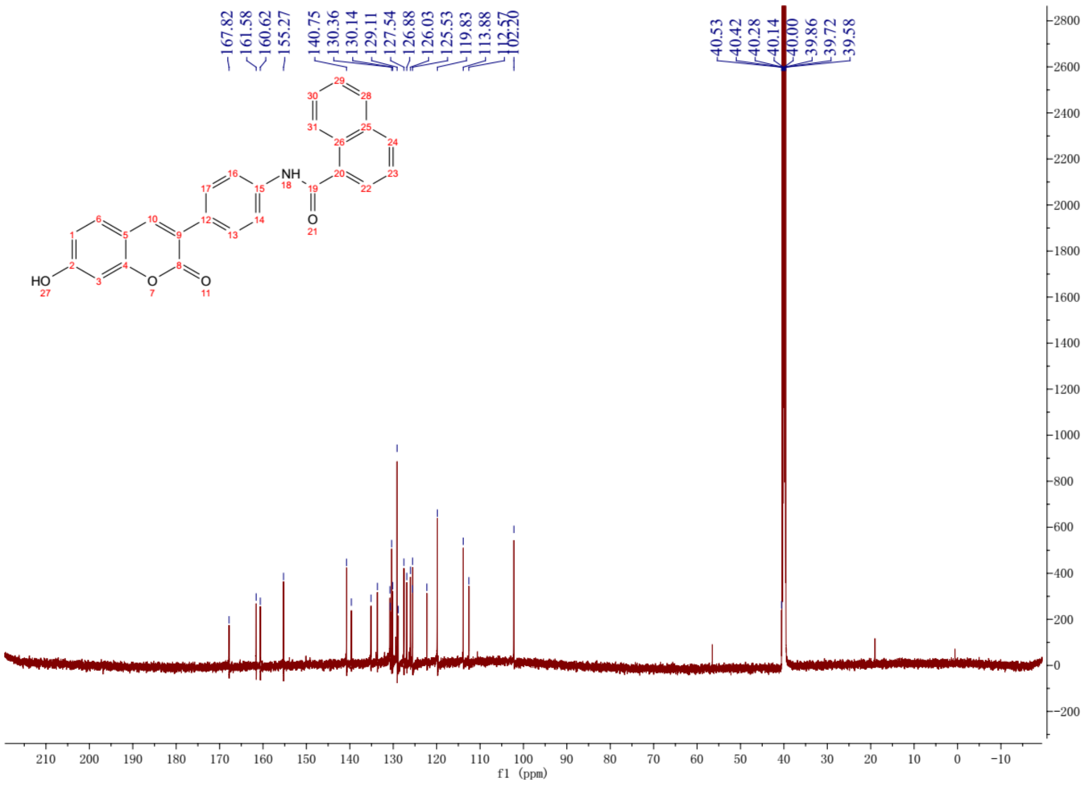

compound **6b** ^{1}HNMR

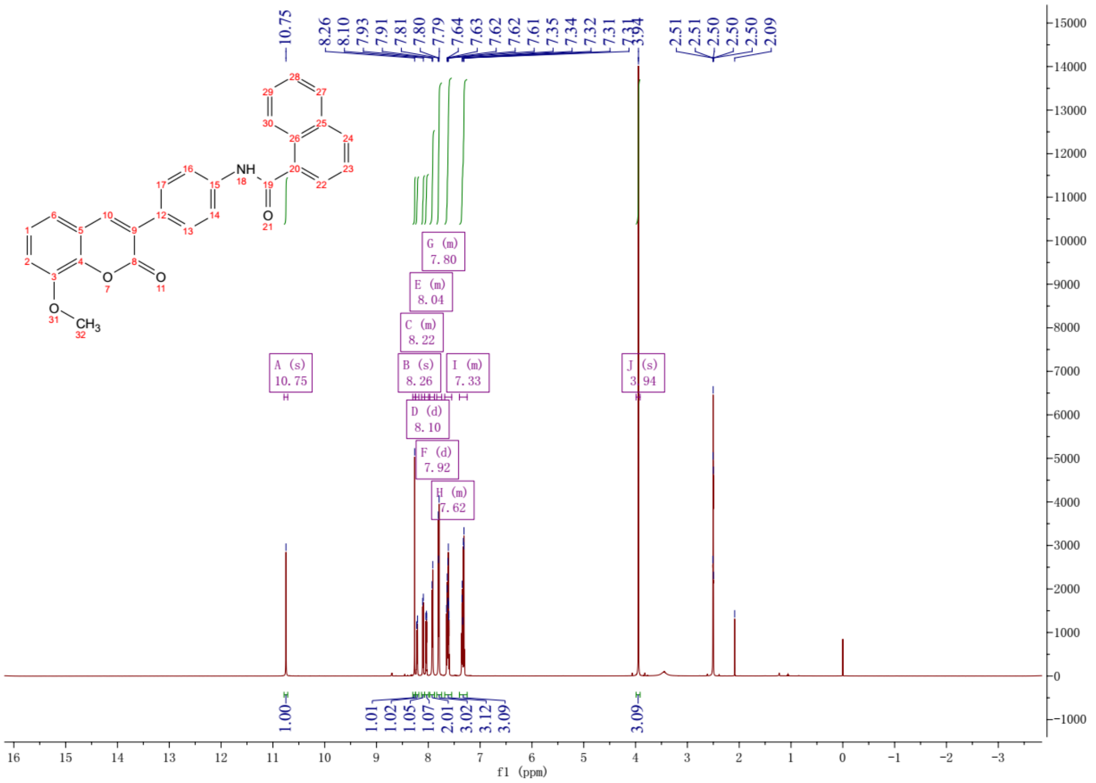

compound **6b** ^{13}CNMR

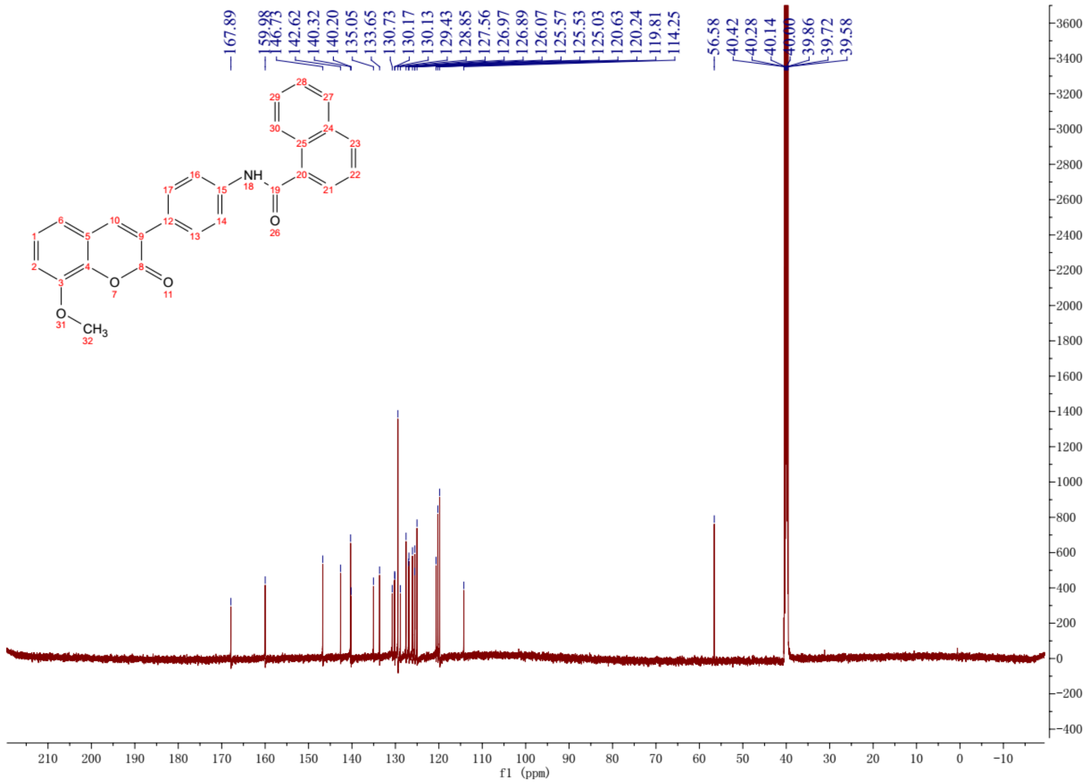

compound **7a** ^{1}HNMR

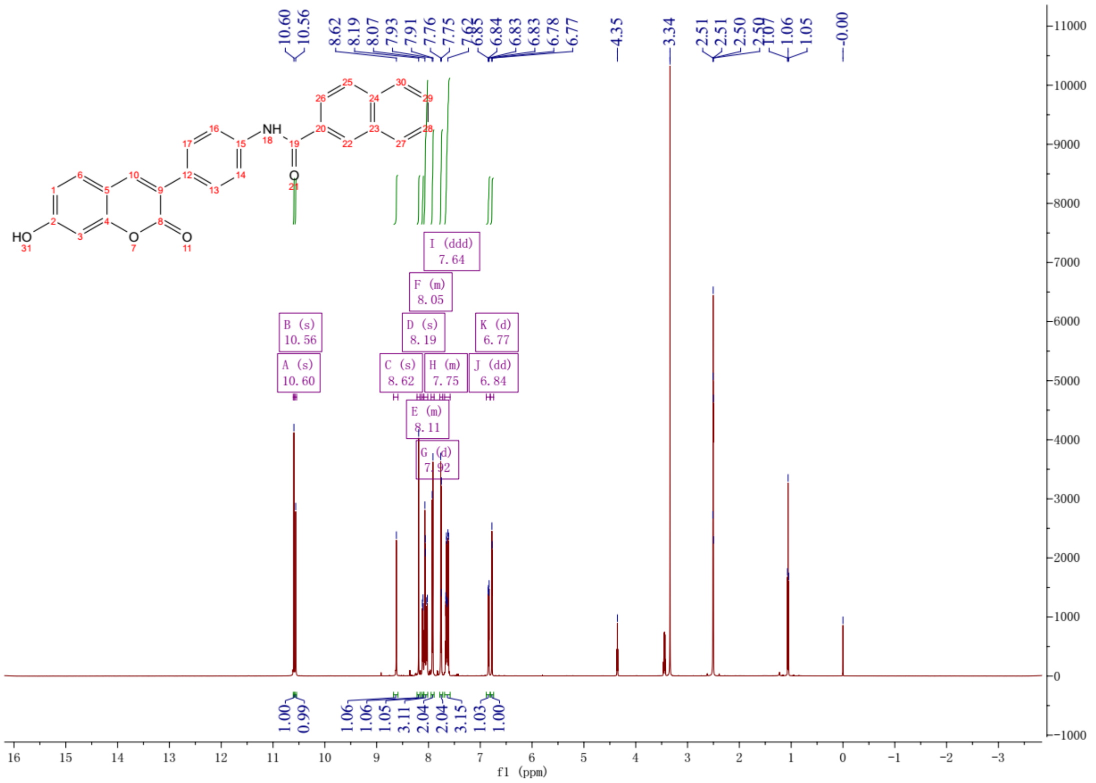

compound **7a** ^{13}CNMR

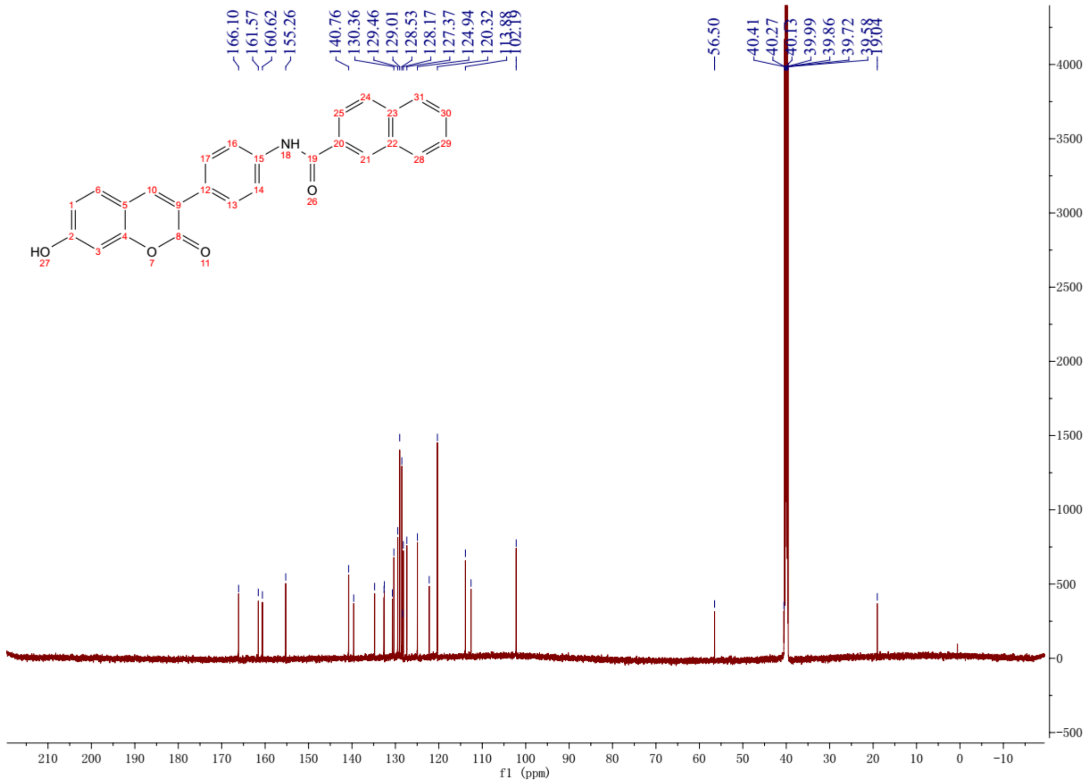

compound **7b** ^{1}HNMR

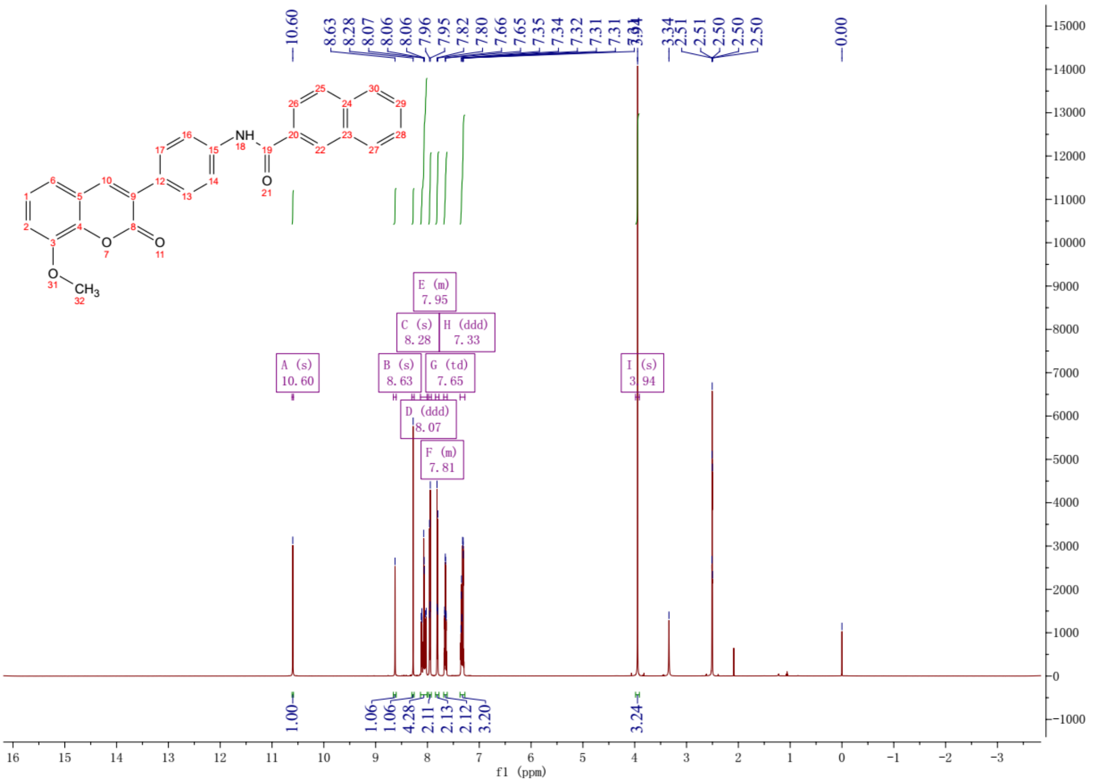

compound **7b** ^{13}CNMR

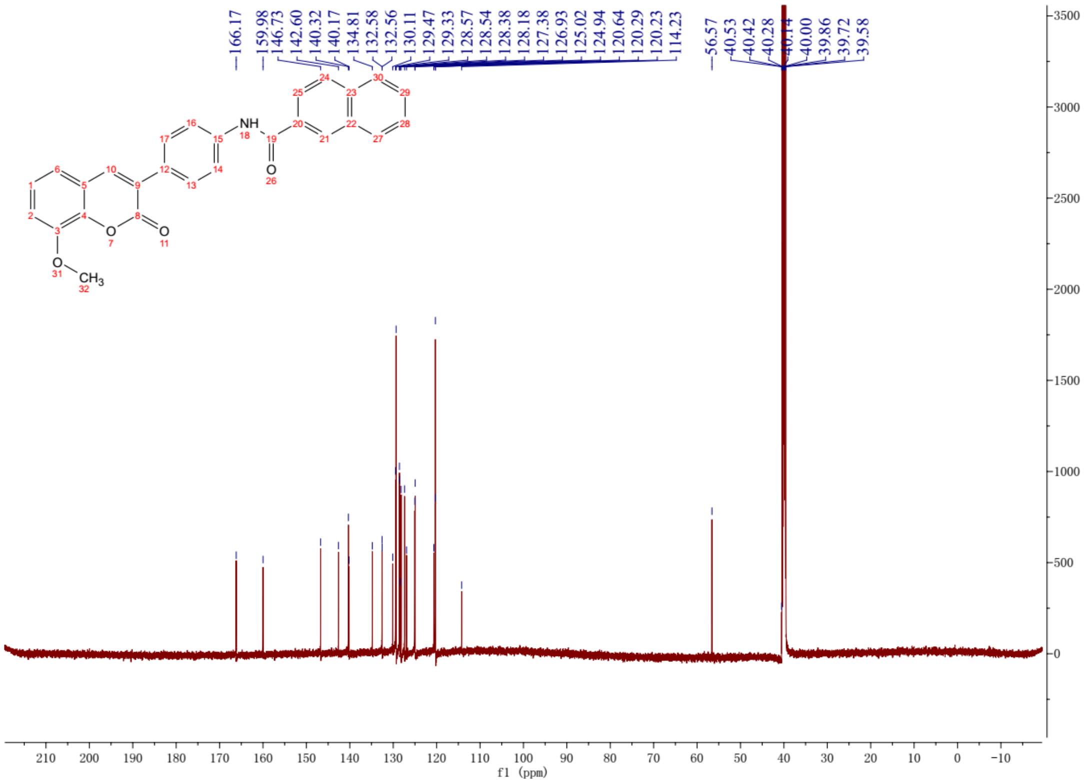

compound **7c** ^{1}HNMR

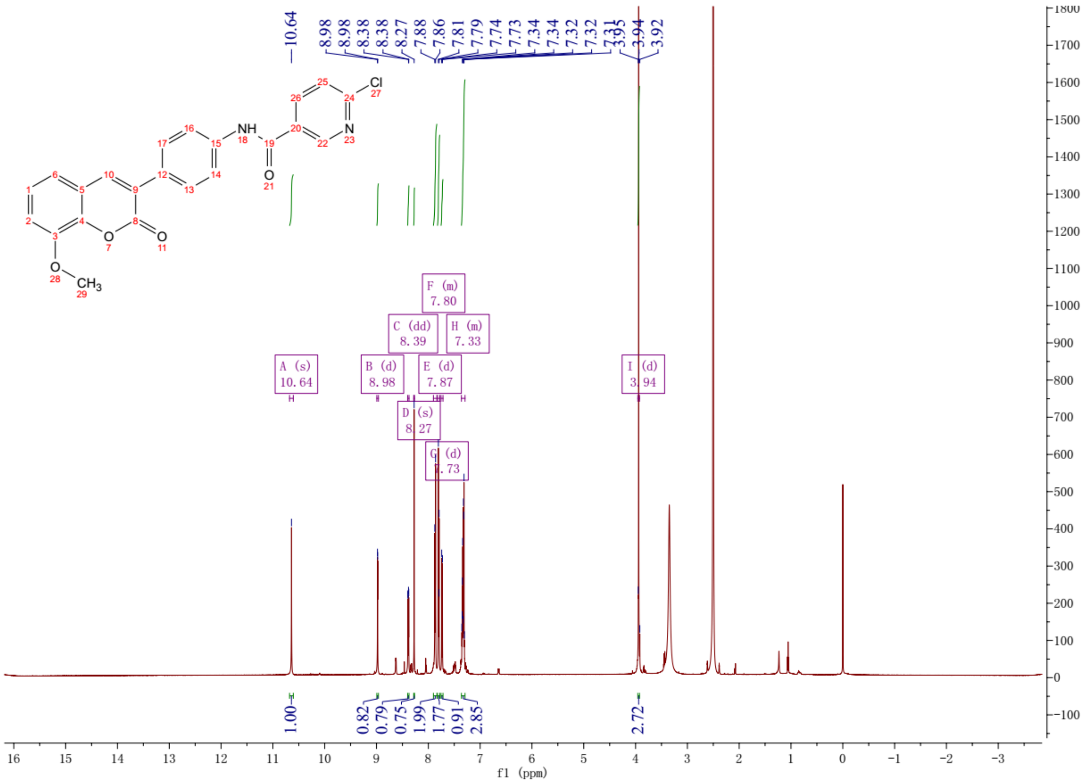

compound **7c** ^{13}CNMR

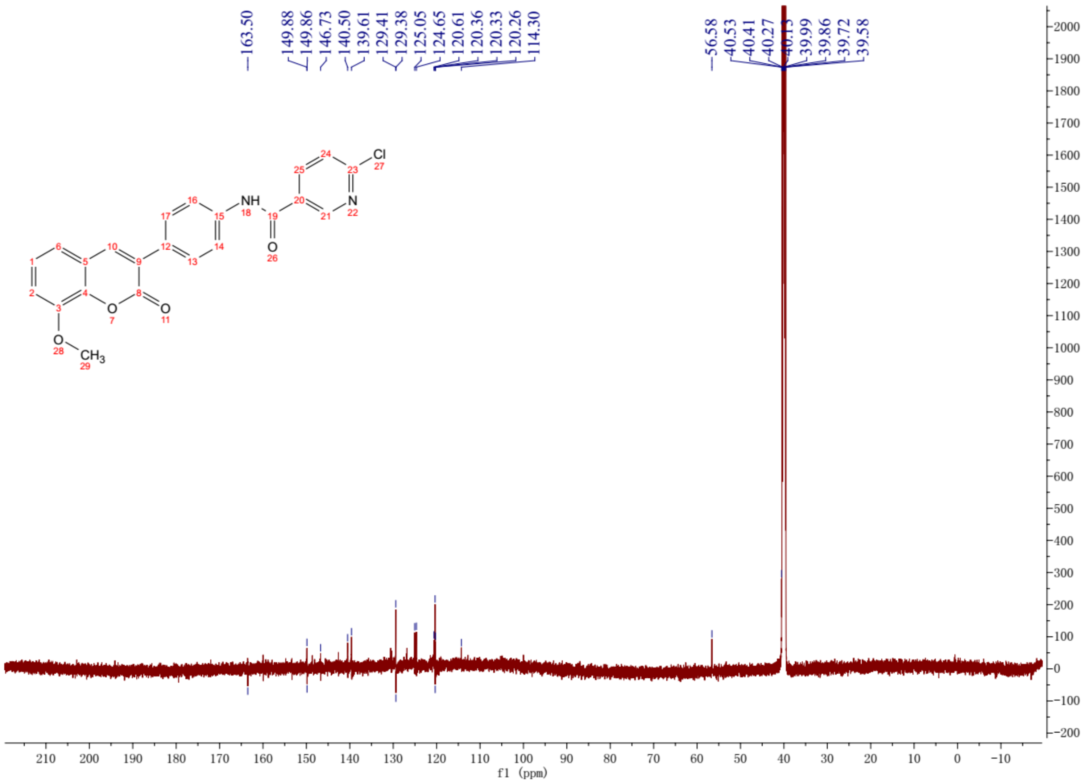

compound **7d** ^{1}HNMR

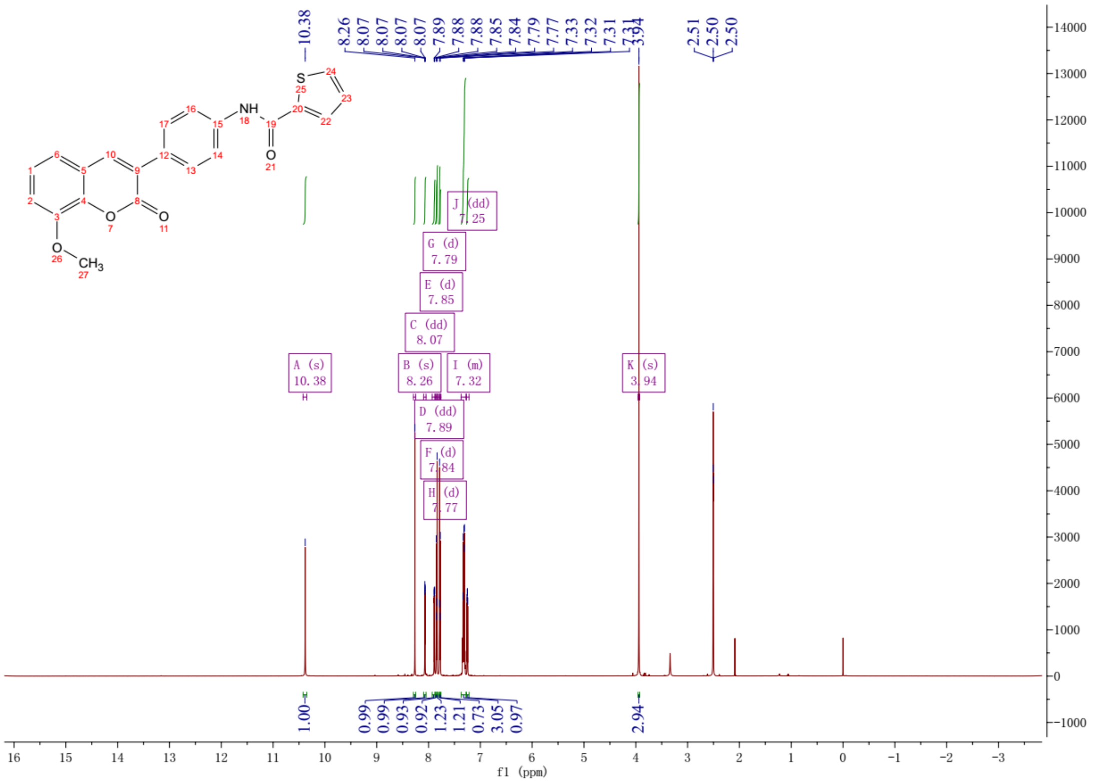

compound **7d** ^{13}CNMR

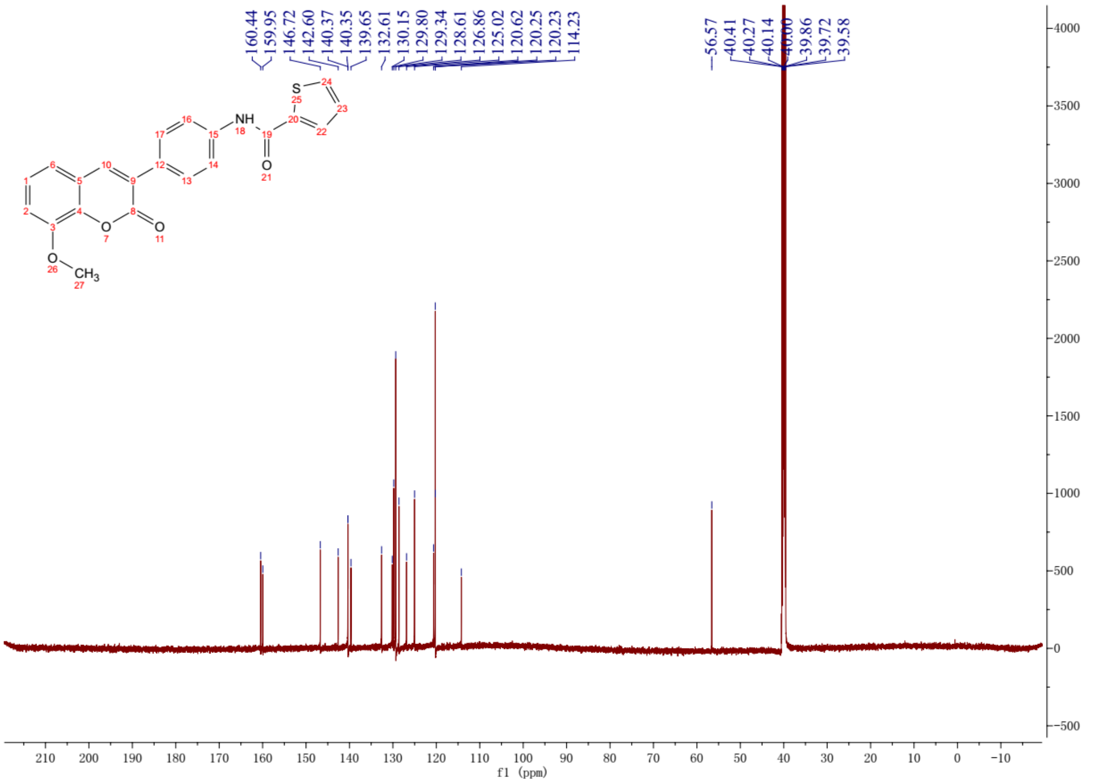

Supplement: Supplemental Material [file IENZ_A_1615484_SM0118.pdf]
